# Introducing chirality in halogenated 3-arylsydnones and their corresponding 1-arylpyrazoles obtained by 1,3-dipolar cycloaddition†

**DOI:** 10.1039/d0ra02368j

**Published:** 2020-04-21

**Authors:** Marcel Mirel Popa, Sergiu Shova, Madalina Hrubaru, Loredana Barbu, Constantin Draghici, Florea Dumitrascu, Denisa E. Dumitrescu

**Affiliations:** Center of Organic Chemistry “C. D. Nenitzescu”, Romanian Academy Spl Independentei 202B 060023 Bucharest Romania fdumitra@yahoo.com; “Petru Poni” Institute of Macromolecular Chemistry Aleea Gr. Ghica Voda 41A Iasi 700487 Romania; Faculty of Pharmacy, Ovidius University Constanta Str. Cpt. Av. Al. Serbanescu 6, Campus Corp C Constanta 900470 Romania

## Abstract

New 1-arylpyrazoles substituted with halogen atoms (Br, I) were synthesized from the corresponding sydnones by 1,3-dipolar cycloaddition. By introduction of a prochiral group such as isopropyl, in the *ortho* position of the benzene ring, in the starting phenylglycine 1 the rotamers caused by the hindered rotation between the phenyl and the heterocyclic ring were detected by NMR spectroscopy for 1-arylpyrazoles and for the first time for 3-arylsydnones. The *N*-nitrosophenylglycines present *E*–*Z* stereoisomerism due to the partial C–N double bond character. All the new compounds were structurally characterized by NMR spectroscopy and confirmed by X-ray crystallography. The crystal structures of *N*-nitrosophenylglycine 2c and of the sydnone 3c present similar Br⋯Br type II halogen contacts.

## Abstract 2

New 1-arylpyrazoles substituted with halogen atoms (Br, I) were synthesized from the corresponding sydnones by 1,3-dipolar cycloaddition.

## Graphical abstract

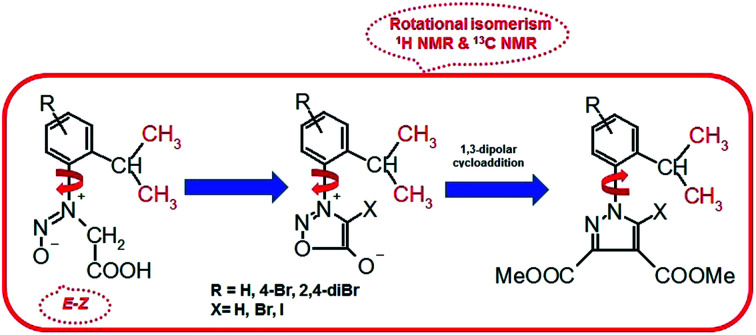

## Introduction

Mesoionic compounds^1–5^ are versatile intermediates in the synthesis of bioactive pyrrole and pyrazole derivatives.^6–11^ Sydnones^12,13^ in particular, have been employed as 1,3-dipoles in obtaining *N*-arylpyrazoles by 1,3-dipolar cycloaddition reaction.^14–28^*N*-Arylpyrazole derivatives have been shown to possess antithrombotic,^29^ anticancer,^30^ parathyroid dysfunction,^31^ antibiofilm,^32^ and antipain^33^ activity which makes them valuable targets for the pharmaceutical field.

Chirality is important in drug-target recognition processes^34^ and atropisomerism could be a useful tool for generating chiral drugs^34^ by blocking the free rotation about a bond axis through steric factors. The most studied systems are biaryls.^35^ The structure of *N*-arylpyrazoles implies the existence of axial chirality when the right substituents are grafted on their framework. By fine tuning of the substituents on the aryl or pyrazole rings the free rotation about the bond axis between the two rings could be hindered and thus leading to the existence of the two rotational isomers. Depending on the value for the energy of the free rotation about the two rings, the enantiomers can be detected by NMR spectroscopy or even the physical separation of the conformational isomers could be achieved. However, even in the case the free energy of rotation is smaller and the atropisomers are not easily separable in normal conditions but instead rotamers are detectable, the conformational analysis is still an important thing to be investigated under de drug-target interaction paradigm, for example.^36^

Herein we present the synthesis of new halogenated 1-arylpyrazoles bearing in mind that halogen atoms could enhance their bioavailability^37^ due to halogen bonding^38^ formation with specific nucleophilic sites in proteins. The introduction of axial chirality in the new 1-arylpyrazoles was pursued and investigated during the synthesis steps starting with the *N*-nitrosoderivatives and sydnone intermediates. The structures of the compounds were confirmed by X-ray single crystal diffraction analysis.

## Results and discussions

### Synthesis and characterization of *N*-nitrosophenylglycines 2

**Scheme 1:**
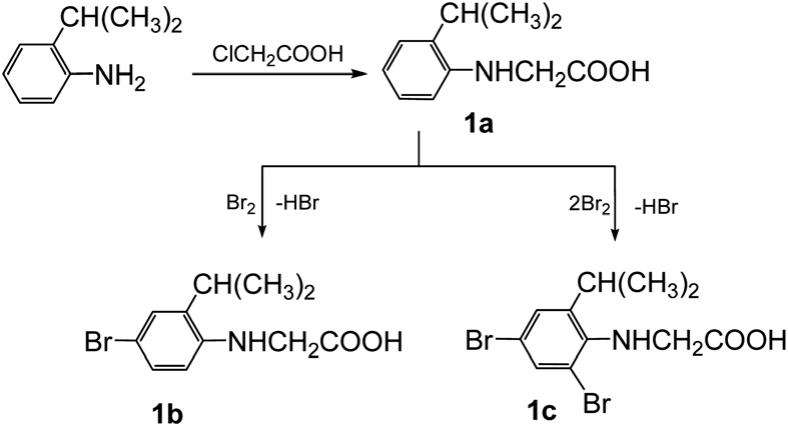
The synthesis of the starting *N*-arylglycines 1a–c.

The synthesis of the 3-arylsydnones was achieved from corresponding *N*-nitroso-*N*-(2-isopropylphenyl)glycines obtained according to literature procedures.^16^ The starting *N*-arylglycine 1a (Scheme 1), precursor of the *N*-nitrosoglycines, was obtained from 2-isopropylaniline by condensation with monochloroacetic acid. Compound 1 was further brominated with Br_2_ to yield the *N*-arylglycines 1b,c (Scheme 1).

**Scheme 2:**
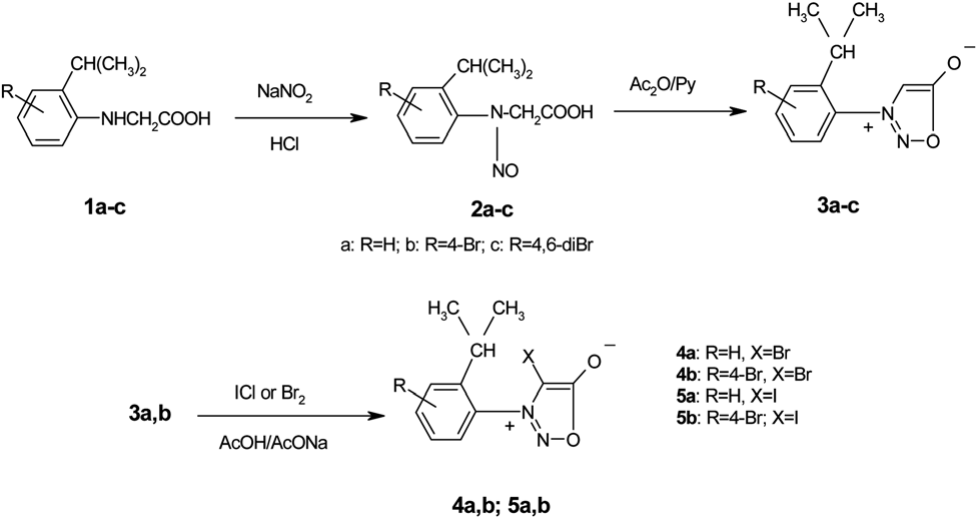
The synthesis of nitrosoaminoacids 2a–c and subsequent synthesis of the halogenated *N*-arylsydnones 3–5.

The ^1^H NMR spectra of the *N*-arylglycines 1a–c confirmed their structure. The isopropyl moiety appears as a doublet at 1.23–1.28 ppm with *J* = 6.8 Hz and a sextet at 2.96 ppm with the same value of the coupling constant. The aromatic region of the ^1^H NMR spectra of the compounds 1a–c presents the expected signals with the characteristic multiplicity (see Experimental section). The nitrosation of *N*-arylglycines 1a–c was made with NaNO_2_ in presence of hydrochloric acid according to Scheme 2. The nitroso derivatives 2a–c were obtained as crystalline solids in up to 90% yield.

From a careful examination of the NMR spectra of the nitrosoderivatives 2a–c we concluded that these compounds were obtained as mixtures of *E*–*Z* isomers which were unquestionably observed at low temperatures due to the fact that in normal conditions the reaction leads to the most stable isomer as will be discussed further. The *E*–*Z* isomerism appears in nitrogen containing compounds in which C–N (*i.e.* amides, oximes, *etc*) or N–N (*i.e.* nitroso) presents partial double bond character due to conjugation.^39–44^ However the *E*–*Z* isomerism in the case of nitrosoacids like 2a–c was less investigated. The activation parameters of the *E*–*Z* inter-conversion in the case of nitrosoamines were investigated by dynamic NMR techniques.^45,46^

**Fig. 1:**
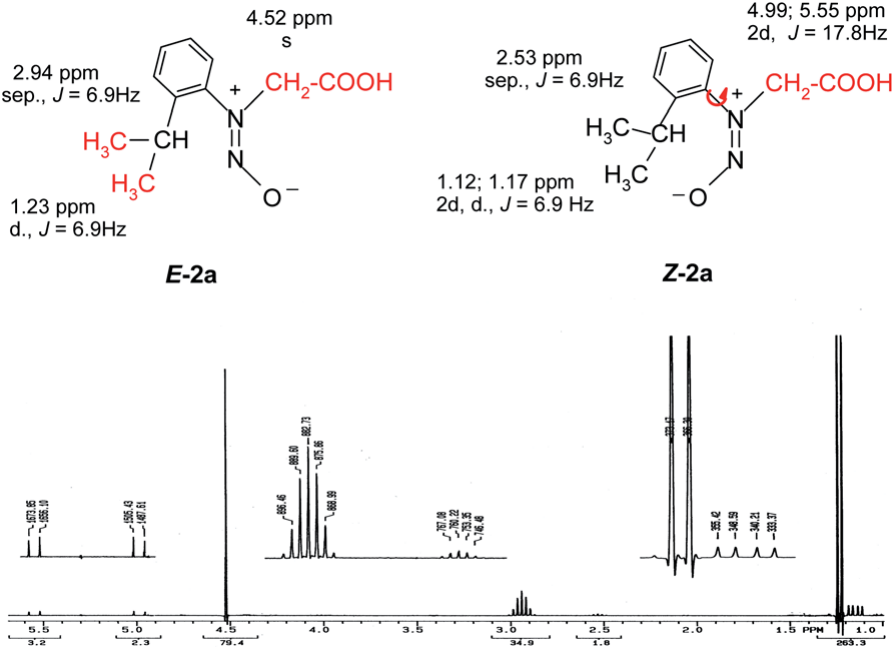
The ^1^H NMR spectrum of 2a showing the presence of both the *Z*–*E* isomers and the atropisomerism of the isomer *Z*-2a depicted by the signal multiplicities of the methyl and methylene protons.

Also the *E*–*Z* configuration was attributed on the rationale that the most voluminous substituent must be in *cis* position in respect to the oxygen atom in the nitroso group. The ^1^H NMR spectrum of the compound 2a shows that the signals of the protons in the isopropyl moiety and the protons in methylene attached to the nitrogen atom appear as two distinctive sets with the integral ratio of 95 : 5 (Fig. 1). For each distinctive sets of signals the multiplicities are different: the methylene attached to the N atom appears as a singlet (for the 95% isomer) and as two doublets forming an AB system with *J* = 17.8 Hz for the 5% isomer. The same is valid for the isopropyl moiety, Table 1 presenting the signals in the aliphatic region and their corresponding multiplicities. The presence of two conformers is observed for *Z*-2a (Fig. 1) due to the hindered rotation about the C–N bond induced by the bulky isopropyl group and the contribution of the nitroso moiety. The different chemical shifts of the analogous atoms in the two rotamers are slightly different due to the magnetic anisotropy of the nitroso group which is in a different spatial relation with the interacting hydrogen atoms.

**Table 1:** The ^1^H NMR signals for the relevant protons in 2b and 2c showing the specific multiplicity characteristic to the *E*–*Z* isomers and the presence of the stable conformers of *Z*-2b and *E*-2c and *Z*-2c detectable in the NMR timescale at room temperature

Isomer	CH_3_, *δ* (ppm); *J* (Hz)	CH, *δ* (ppm); *J* (Hz)	CH_2_, *δ* (ppm); *J* (Hz)	H-6′	%
*E*-2b	1.22, d, 6.9	2.90, sep., 6.9	4.49, s	7.28, s	95
*Z*-2b	1.10, 1.16, 2d, 6.8	2.49, sep., 6.8	4.94, 5.53, 2d, 17.8	6.95, s	5
*E*-2c	1.18, 1.21, 2d, 6.9	3.38, sep., 6.9	3.95, 4.94, 2d, 16.8	—	58
*Z*-2c	1.10, 2d, 6.8	2.82, sep., 6.8	4.91, 5.78, 2d, 17.8	—	42

The *Z*–*E* isomerism was also observed for compounds 2b and 2c. For 2b the protons in the aromatic ring, present two sets of signals corresponding to the two stereoisomers (Table 1) while H-6′ presents different chemical shifts also due to the spatial influence of the nitroso group.

**Fig. 2:**
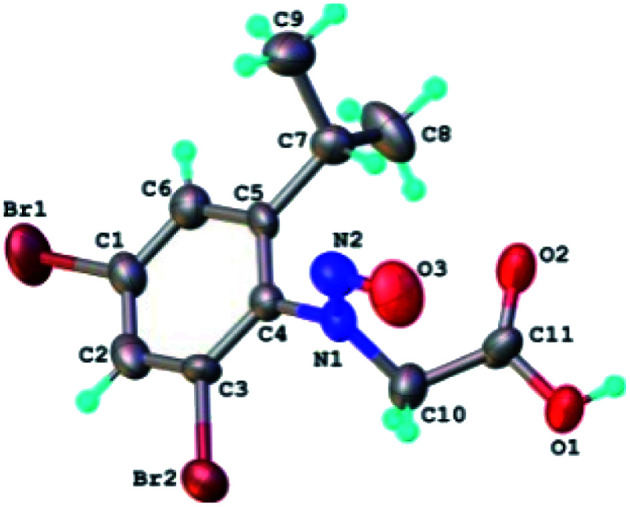
X-ray molecular structure of compound 2c with atom labeling and thermal ellipsoids at 50% level.

The non-equivalence of the methyl protons of the isopropyl moiety suggests that *Z*-2a and *Z*-2b present axial isomerism which is induced by the hindered rotation about the C–N bond caused by the bulky isopropyl group. This is elegantly confirmed in the case of 2c, in which by introducing the bromine atom in the *ortho* position of the aryl ring with respect to the nitroso group gives the two *E*–*Z* stereoisomers in 58 : 42 ratio observed by ^1^H NMR spectroscopy (Fig. 1S†). Both *Z*-2c and *E*-2c present the coexistence of two stable conformers in solution (Table 1). The NMR studies indicated that *Z*-2c transforms in *E*-2c in solution similar to 2a and 2b. Thus one can conclude that *E*-2c is the thermodynamically stable stereoisomer and *Z*-2c is obtained by the nitrosation reaction of 1c to 2c which goes under kinetic control. We were able to separate *E*-2c from the mixture and to characterize it separately by NMR (Fig. 2S†) spectroscopy (see ESI†) and by single crystal X-ray diffraction analysis.

The structure of the *N*-nitrosophenylglycines 2 was investigated by X-ray single crystal diffraction of 2c as representative compound. Suitable crystals of 2c were grown by slow evaporation from acetonitrile. The X-ray diffraction analysis (Fig. 2) confirms the structure of 2c as being the *E* stereoisomer in solid state. The geometric parameters are listed in Table 2S (ESI).†

**Fig. 3:**
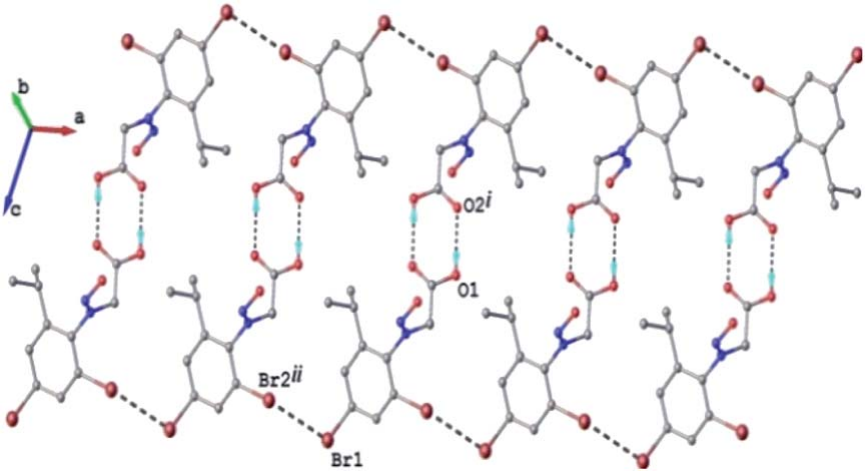
1D supramolecular network in the crystal structure 2c. H-atoms not involved in hydrogen bonding are omitted. H-bonds and Br⋯Br contacts are shown in dashed-black lines.

The crystal structure of 2c can be characterized as the parallel packing of the discrete infinite ribbons running along a crystallographic axis (Fig. 3S†). The structure of one-dimensional ribbon-like supramolecular architecture is shown in Fig. 3 showing short Br⋯Br contacts. The value of the intermolecular Br⋯Br distance, 3.536(1) Å is under the sum of van der Waals radii (3.70 Å). Also the values of the angles C–Br⋯Br are 113.0° and 173.9°, respectively.

The Br⋯Br contacts are by definition included in the class of the halogen bonds of type II.^47^

The energy of the cyclic dimer made by the hydrogen bond in the carboxylic acid was calculated at HF/3-21 level as implemented in Crystal Explorer 17.^48,49^ The calculated value of −73 kJ mol^−1^ place the O–H⋯O=C bond strength as usual for such kind of compounds.^50^

### Synthesis and characterization of the 3-arylsydnones 3–5

The new nitrosophenylglycines 2a–c were used further in the synthesis of the *N*-arylsydnones 3a–c (Scheme 2). The new sydnones were obtained in good yields (see ESI†). The sydnones 3a,b were then halogenated in position 4 to give 4-bromosydnones 4a,b and 4-iodosydnones 5a,b by using iodine monochloride or molecular bromine. Unfortunately the halogenation in the case of 3c did not give satisfactory results owing to prolonged reaction time or required higher temperatures which led to the decomposition of the reaction products.

The structure of the new compounds 3–5 (Table 2) was investigated and confirmed by NMR spectroscopy. The investigation of the NMR spectra showed that 1-arylsydnones 3c and 4b and 5a,b present also stable rotamers induced by the hindered rotation between the phenyl and sydnone ring given by the bulky isopropyl moiety and the voluminous halogen atom in position 4 of the sydnone ring in the case of 4 and 5. The diastereotopic isopropyl group has been used to probe the axial chirality which could be studied by NMR spectroscopy due to the magnetic non-equivalence of the methyl protons and carbon atoms caused by the hindered rotation around the C–N bond.

**Table 2:** The structures and features of the new halogenated 3-arylsydnones 3–5

No.	R	X	Yield (%)	Mp (°C)
3a	H	H	87	68–69
3b	4-Br	H	72	100–101
3c	4,6-Dibromo	H	54	169–171
4a	H	Br	85	91–92
4b	4-Br	Br	82	142–144
5a	H	I	82	179–180
5b	4-Br	I	82	195–197

**Fig. 4:**
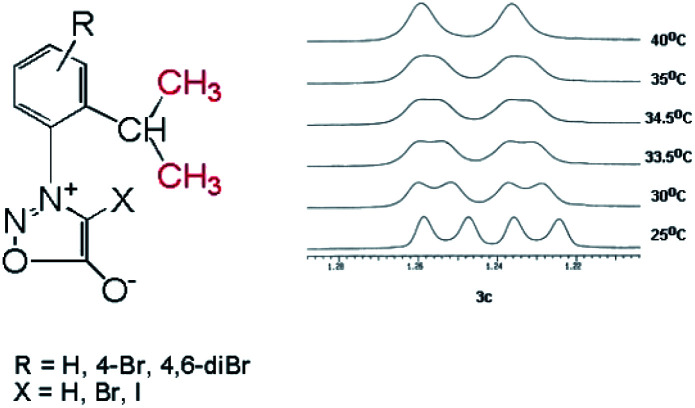
The two methyl protons in the isopropyl group become anisochronous due to the hindered rotation about the C–N group. The ^1^H NMR signals of the two methyl protons in 3c which coalesce at ∼35 °C.

In the case of the sydnones 3a–c, we could detect the presence of the two rotamers at room temperature, only in the case of 3c which possess a Br atom attached to the other *ortho* position of the phenyl ring in respect to the sydnone and contributes by its volume to the steric hindrance between the phenyl and sydnone rings. Thus, in the ^1^H NMR spectrum of 3c the methyl protons of the isopropyl appear as two doublets at room temperature (Fig. 4) while the ^13^C NMR spectrum presents two distinctive signals for the non-equivalent methyl carbon atoms.

In the case of 4-bromosydnones 4a,b without a bromine in the position 6′ of the benzene ring the ^1^H NMR spectra does not provide relevant data regarding the presence of axial chirality at room temperature but the ^13^C NMR spectrum present the methyl groups C atoms as a broad singlet showing that at room temperature the energy barrier for the free rotation about the C–N bond is reached. Comparing the ^1^H and ^13^C NMR spectra of compounds 3c and 4a,b one can conclude that the presence of a Br atom on the *ortho* position of the phenyl ring has a greater influence upon the free rotation than in the case it is attached to the 4 position of the sydnone ring as the presence of the two rotamers is clearly distinguished in solution. For the 4-iodosydnone 5a,b the volume of iodine atom has an influence upon the signals of the methyl groups in the isopropyl moiety, the two methyl groups appearing as two distinct doublets in dilute solutions in ^1^H NMR. The ^13^C NMR spectra show also two distinct signals for the methyl groups. The free rotation energy barrier about the single bond must be therefore a little higher than in the analogous 4-brominated sydnones but still the free rotation has an energy sufficiently low to make to separate them in usual conditions.

**Fig. 5:**
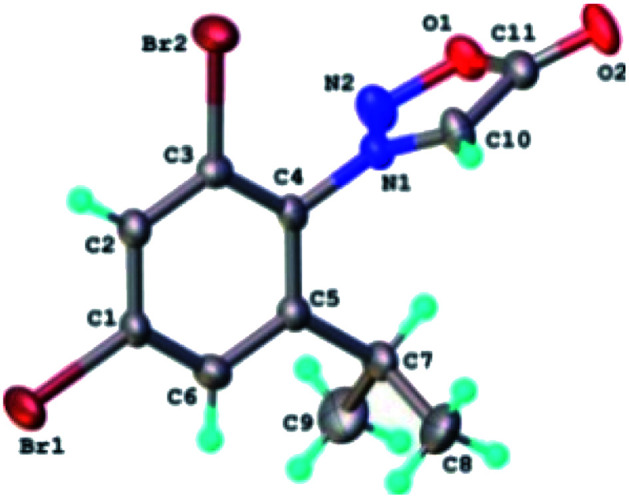
X-ray molecular structure of compound 3c with atom labeling and thermal ellipsoids at 50% level.

The results of X-ray diffraction investigation for 3c is depicted in Fig. 5, while bond distances and angles are summarized in Table 2S.† This compound exhibits a molecular crystal structure resulting from the packing of two-dimensional supramolecular double-layer in parallel orientation (as shown in Fig. 4S†). The self-assembling of two-dimensional layer occurs through the weak H-bonds where C_2_–H group of the phenyl acts as donor towards carbonyl oxygen atom O_2_ of adjacent molecule as acceptor of proton (Fig. 4S†).

**Fig. 6:**
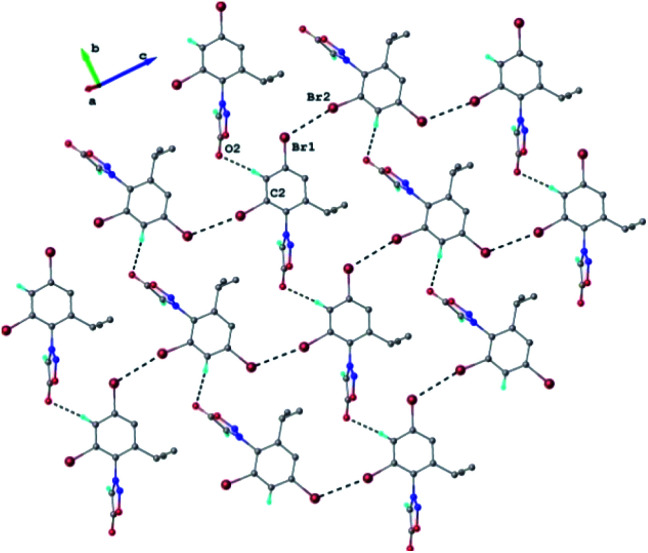
2D supramolecular network in the crystal structure 3c. H-atoms not-involved in hydrogen bonding are omitted. H-bonds and Br⋯Br contacts are shown in dashed-black lines. H-bond parameters: C_2_–H⋯O_2_ [C_2_–H 0.93 Å, H⋯O_2_ 2.54 Å, C_2_⋯O_2_(*x*, 1 − *y*, *z*) 3.359(5) Å, ∠C_2_HO_2_ 147.1°].

Each double-layer architecture is built-up from two 2D networks (Fig. 6), which is consolidated due to the C–H⋯O hydrogen bonding and halogen⋯halogen interactions at 3.6020(8) Å for Br⋯Br′ distance (Fig. 6). In the sydnone 3c the intermolecular distance Br⋯Br 3.6020(8) is also under the value of vdW and values of the angles C–Br⋯Br of 119.57° and 169.39° respectively. The torsion angle between the plane of the phenyl ring and the plane of the sydnone ring has the value of 86.5°.

Beside the halogen Br⋯Br contacts in 3c we observed that 4-iodosydnones presented interesting halogen bonding affinity in solution^51^ in presence of different bases, and thus we envisaged that halogenated sydnones could provide ideal templates for studying the halogen bonding either in solution or solid state which will aim to investigate further.

### Synthesis and characterization of the 1-arylpyrazoles 6a–g

**Scheme 3:**
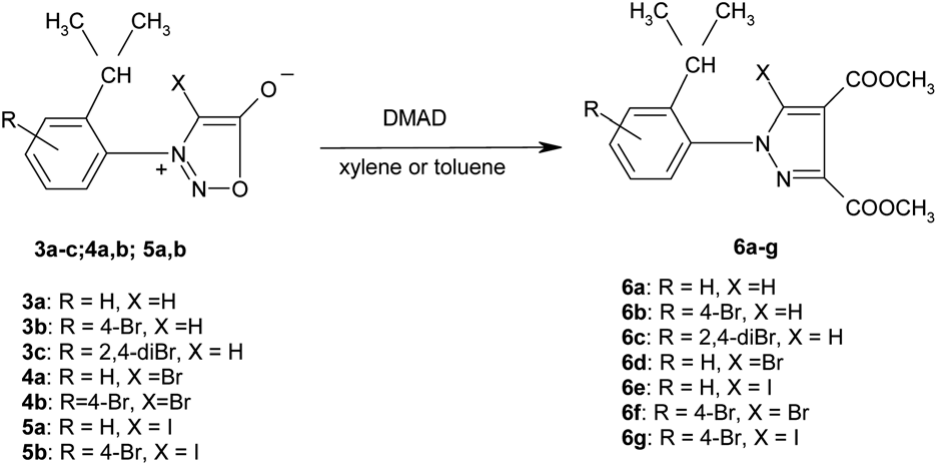
The synthesis of the halogenated 1-arylpyrazoles 6a–g.

The new halogenated 3-arylsydnones were employed in the final step in the synthesis of new halogenated 1-arylpyrazoles using the 1,3-dipolar cycloaddition reaction with activated acetylenic dipolarophiles. Thus, the new compounds 6a–g were obtained starting from the sydnones 3–5 at reflux temperature in toluene or xylene as solvent (Scheme 3).

**Scheme 4:**
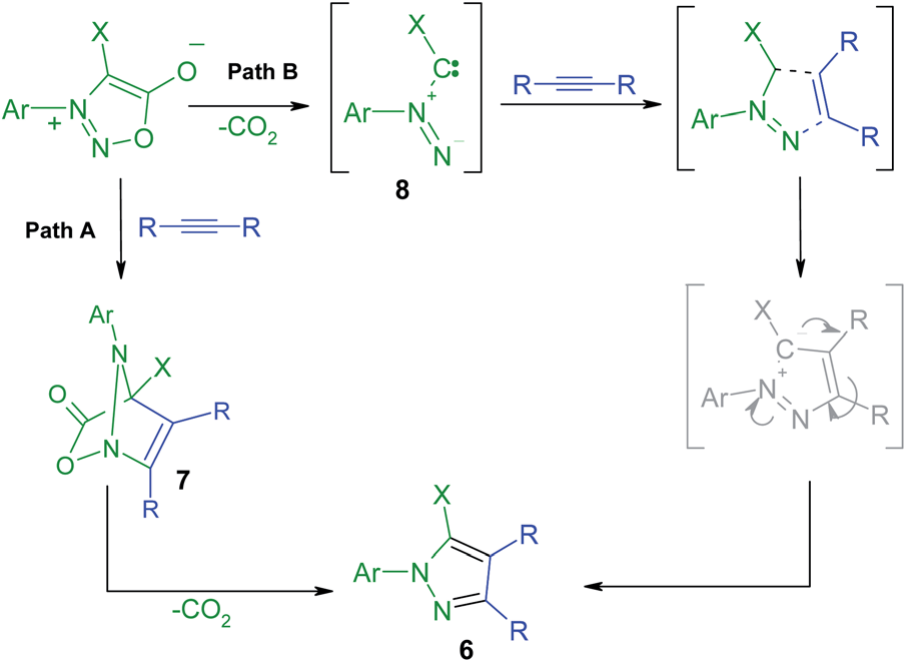
Proposed mechanism pathways for the formation of the pyrazoles 6 by formal [3 + 2] cycloaddition of DMAD to sydnones.

The mechanism of the [3 + 2] addition of the DMAD to sydnones was investigated^28^ and generally accepted as going through a formation of a bicyclic intermediate 7 as presented in the Scheme 4 which leads to the final pyrazole compounds by elimination of one CO_2_ molecule and further aromatization (Path A). Literature data^52,53^ suggest also another mechanism implying an acyclic intermediate of type 8 (Path B). In the case of the sydnones such intermediate was demonstrated to coexist to a lesser extent with the bicyclic one, giving pyrazoles with another substitution pattern, a 1,3-dipole structure being suggested.^27^

Table 3 presents the new synthesized pyrazoles 6a–g and their features.

**Table 3:** The new 1-arylpyrazoles 6a–g

No.	R	X	Yield (%)	Mp (°C)
6a	H	H	87	62–63
6b	4-Br	H	94	139–140
6c	2,4-DiBr	H	67	113–115
6d	H	Br	87	92–93
6e^52^	H	I	82	156–157
6f	4-Br	Br	86	103–104
6g	4-Br	I	80	128–131

**Fig. 7:**
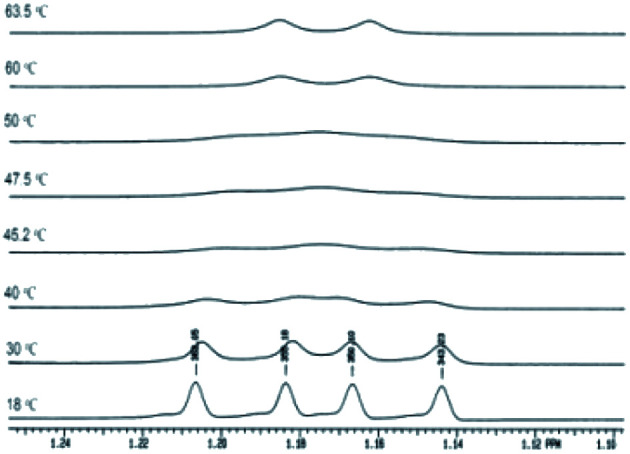
The ^1^H NMR signals of the methyl groups in the isopropyl moiety for compound 6e.

Compounds 6a–g where characterized by NMR spectroscopy and their structure was confirmed (Experimental section). The singlet in the range 8.03–8.06 belongs to H-5 proton in the pyrazole moiety while the protons in the phenyl group are in accordance with the substitution pattern. In the compounds 6a,b,d the two methyl groups in the isopropyl moiety appear as doublets due to the coupling with the adjacent methine hydrogen. In compounds 6c the presence of the bromine atom in the *ortho* position the phenyl ring is sufficient to increase the rotation barrier of the two rings about the single bond. This is reflected in the ^1^H NMR spectrum by the magnetic non-equivalence of the two methyl groups which appear as two doublets at 1.12 and 1.19 ppm (Fig. 7). In the ^13^C NMR spectra the methyl groups in the isopropyl moiety appear as a singlet for 6a,b,d and for 6c the spectrum shows two different signals highlighting the magnetic non-equivalence of the two protons in discussion. For the 5-bromopyrazoles 6d,f the most important proof of the hindered rotation is the ^13^C NMR which shows two signals for the methyl groups while the proton NMR being more sensible to dilution shows only one doublet. For the 5-iodopyrazoles 6e,g the magnetic non-equivalence of the two methyl groups is clear in both ^1^H and ^13^C NMR spectra. The NMR data suggests that the most effective hindrance of the free rotation about Ar–N bond is when the halogen atom and the isopropyl group are both grafted on the same ring, the phenyl ring in particular.

The compound 6e was studied by dynamic NMR and the resulted energy barrier of rotation was calculated at a coalescence temperature of 50 °C using the Gutowski relation. The energy was calculated to be 70.5 kJ mol, which is high enough to try the separation of the two enantiomers.

**Fig. 8:**
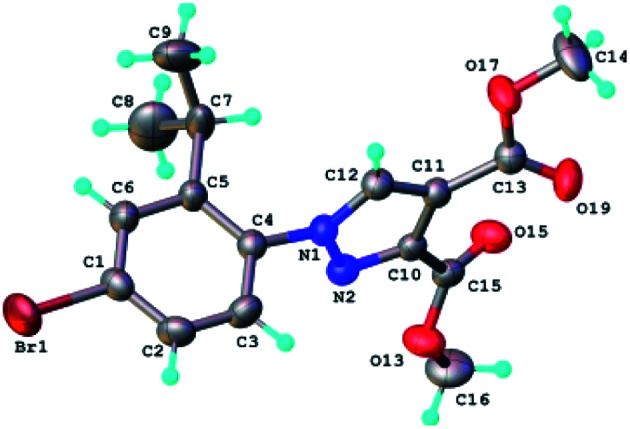
X-ray molecular structure of compound 6b with atom labeling and thermal ellipsoids at 50% level.

The structure of the highly substituted pyrazoles has been also confirmed by single crystal X-ray diffraction analysis. The molecular structure of the representative compound 6b is shown in Fig. 8 and bond distances and angles in Table 1S.†

The analysis of the crystal packing shows the presence of two-dimensional supramolecular layers, which are formed by a system of intermolecular C–H⋯O hydrogen bonding. A view of this layer is depicted in Fig. 5S.† The crystal structure essentially results from the packing of these layers parallel to 011 plane, as shown in Fig. 6S.† The torsion angle between the plane of the phenyl ring and the plane of the pyrazole ring has the value of 75.57°.

We have investigated recently the propensity of halogen bonding in halogenated 1-arylpyrazoles and we found them as versatile structures for study of this interesting interaction.^54^

## Conclusions

In conclusion, new halogenated 3-(2-isopropylphenyl)sydnones and 1-(2-isopropylphenyl)pyrazoles were obtained from the corresponding *N*-nitrosoderivatives. The bulky halogen atoms on the phenyl or the five membered rings (sydnone and pyrazole) and the presence of the isopropyl on the *ortho* position of the phenyl ring induced a hindered rotation about the C–N bond, two stable rotamers being confirmed by NMR spectroscopy in solution. The X-ray single crystal analysis confirmed the structure of the new compounds and highlighted their conformation in solid state. The X-ray diffraction confirmed the structure of the *E*-2c*N*-nitrosophenylglycine and showed similar Br⋯Br short contacts with sydnone 3c. All the halogenated sydnones and pyrazoles are to be further investigated regarding their propensity for halogen bonding as the solution studies and X-ray diffraction analysis showed promising results.

## Experimental

### The synthesis of *N*-(2-isopropyl)phenylglycine (1a)

Mp 139–141 °C. Yield 46%. 30 mL (0.21 mol) of 2-isopropylaniline are mixed with 17 g (0.18 mol) of monochloroacetic acid in 200 mL water and 10 mL ethanol. The reaction mixture is kept under reflux for 3 h. The reaction mass is cooled with water and then stirred for 1 h on an ice bath. The formed precipitate is removed by filtration and then washed with water and with benzene after drying to remove any unreacted 2-isopropylaniline. Anal. for C_11_H_15_NO_2_ (193.2): found: C 68.67; H 8.04; N 7.52. Calcd: C 68.37; H 7.82; N 7.25. ^1^H NMR (CDCl_3_; *δ*, ppm; *J*, Hz): 1.28 (d, 6H, 6.8; Me); 2.96 (sep., 1H, 6.8; CHMe_2_); 4.03 (s, 2H, CH_2_); 6.15 (sl, 2H, COOH, NH); 6.54 (dd, 1H, 7.6; 1.5; H-6′); 6.83 (td, 7.6; 1.7; H-4′); 7.13 (td, 7.6; 1.5; H-5′); 7.19 (dd, 7.6; 1.7; H-3′). ^13^C NMR (CDCl_3_; *δ*, ppm): 22.3 (Me); 27.3 (CHMe_2_); 46.0 (CH_2_); 110.8 (C-6′); 118.9 (C-4′); 125.3 (C-3′); 126.8 (C-5′); 133.0 (C-2′); 143.3 (C-1′); 176.1 (CO).

### The synthesis of *N*-phenylglycines 1b,c

*N*-(2-Isopropyl)phenylglycine 3.9 g (20 mmol) are suspended in 20 mL glacial acetic acid. Over this solution is added by stirring 20 mmol Br_2_ for 1b or 40 mmol Br_2_ for 1c dissolved in 5 mL acetic acid. The reaction is kept under stirring for *cca.* 15–20 min and then the reaction mass is poured over cold water under continuous stirring. The formed precipitate is filtered and washed with water on the filter paper, and then the compound is dried.

#### *N*-(4-Bromo-2-isopropylphenyl)glycine (1b)

White powder crystallized from ethyl acetate. Yield 94%, mp 191–193 °C. Anal. for C_11_H_14_BrNO_2_ (272.14): found: C 48.87; H 5.47; Br 29.76; N 5.42. Calcd: C 48.55; H 5.19; Br 29.36; N 5.15. ^1^H NMR (CDCl_3_ + TFA; *δ*, ppm; *J*, Hz): 1.23 (d, 6H, 6.8; 2CH_3_); 3.25 (sep., 1H, 6.8; C**H**Me_2_); 3.91 (s, 2H, CH_2_); 7.33 (d, 1H, 8.6; H-6′); 7.51 (dd, 1H, 8.6; 2.2; H-5′); 7.64 (d, 1H, 2.2; H-3′); ^13^C NMR (CDCl_3_ + TFA; *δ*, ppm): 23.6 (CH_3_); 27.8 (**C**HMe_2_); 52.5 (CH_2_): 124.2 (C-6′); 124.9 (C-4′); 130.8 (C-2′); 131.3; 131.6 (C-3′; C-5′); 143.7 (C-1′); 168.9 (CO).

#### *N*-(4,6-Dibromo-2-isopropylphenyl)glycine (1c)

White crystals were crystallized from cyclohexane; yield 76%; mp 103–105 °C (desc.). Anal. for C_11_H_13_Br_2_NO_2_ (351.04): found: C 37.97; H 4.06; Br 45.90; N 4.31. Calcd: C 37.63; H 3.73; Br 45.52; N 3.99. ^1^H NMR (CDCl_3_; *δ*, ppm; *J*, Hz): 1.23 (d, 6H; CH_3_); 3.25 (sep., 1H, 6.8; C**H**Me_2_); 3.91 (s, 2H, CH_2_); 7.29 (d, 1H, 2.2; H-3′); 7.53 (d, 1H, 2.2; H-5′); 7.70 (sl, 2H, N**H**, COO**H**). ^13^C NMR (CDCl_3_; *δ*, ppm): 23.7 (2CH_3_); 28.7 (CHMe_2_); 50.3 (CH_2_); 116.7 (C-6′); 119.2 (C-4′); 129.0 (C-3′); 142.0 (C-1); 144.9 (C-1′); 176.7 (CO).

### Procedures for obtaining *N*-nitroso-*N*-phenylglycines

*N*-(2-Isopropylphenyl)glycine 20 mmol are dissolved in 40 mL solution of 5% NaOH and 21.5 mmol of NaNO_2_ are added. Over this mixture 10 mL conc. HCl are added under vigorous stirring and cooling on an ice bath, until the pH will reach a value of 1.5–2. The stirring continues for another 15 min while the nitrosoderivative precipitates. The *N*-nitrosophenylglycine is filtered washed with water on the filter and let to dry in air.

#### *N*-Nitroso-*N*-(2-isopropylphenyl)glycine (2a)

White crystals obtained from ethanol-benzene mixture; yield 94%; mp 104–106 °C (desc.). Anal.: C_11_H_14_N_2_O_3_ (222.24): found: C 59.77; H 6.66; N 12.42. Calcd: C 59.45; H 6.35; N 12.21. ^1^H NMR (CDCl_3_; *δ*, ppm; *J*, Hz): *E* (95%): 1.23 (d, 6H, 6.9; CH_3_); 2.94 (sep., 1H, 6.9; **CH**Me_2_); 4.52 (s, 2H, CH_2_); 7.31–7.44 (m, 2H, H-3; H-5); 7.49–7.53 (m, 2H, H-4; H-6); *Z* (5%): 1.12; 1.17 (2d, 6H, 6.8; CH_3_); 2.53 (sep., 1H, 6.8; **CH**Me_2_); 4.99; 5.55 (2d, 2H, 17.8; CH_2_); ^13^C NMR (CDCl_3_; *δ*, ppm): *E* (95%): 24.0 (CH_3_); 28.2 (**C**HMe_2_); 50.1 (CH_2_); 126.9; 127.3; 130.3 (C-3′, C-4′, C-5′, C-6′); 138.9 (C-1′); 145.4 (C-2′); 171.1 (CO). *Z* (5%): 23.7; 23.9 (CH_3_); 28.6 (**C**HMe_2_); 54.9 (CH_2_); (CO).

#### *N*-Nitroso-*N*-(4-bromo-2-isopropylphenyl)glycine (2b)

The same procedure as for 2a is employed. The compound precipitates as oil which is extracted twice with 15 mL CH_2_Cl_2_. This solution is dried over CaCl_2_ and after the evaporation of CH_2_Cl_2_ the amorphous mass is crystalized from ethyl acetate-benzene. Yield 89%; mp 121–123 °C (desc.). Anal. for C_11_H_13_BrN_2_O_3_ (301.14): found: C 44.09; H 4.72; Br 26.94 N 9.59. Calcd: C 43.87; H 4.35; Br 26.53; N 9.30; ^1^H NMR (CDCl_3_; *δ*, ppm; *J*, Hz): *E*: 1.22 (d, 6H, 6.9; CH_3_); 2.90 (sep., 1H, 6.9; CHMe_2_); 4.49 (s, 2H, CH_2_); 7.28 (d, 1H, 8.4; H-6′); 7.47 (dd, 1H, 8.4; 2.2; H-5′); 7.61 (d, 1H, 2.2; H-3′); 9.45 (sl, 2H, COOH); *Z*: 1.10; 1.16 (d, 6H, 6.9; CH_3_); 2.48 (sep., 1H, 6.9; C**H**Me_2_); 4.94; 5.53 (2d, 2H, 17.8; CH_2_); 6.95 (d, 1H, 8.4; H-6′); 7.37 (dd, 1H, 8.4; 2.2; H-5′); 7.50 (d, 1H, 2.2; H-3′); 9.45 (bs, 2H, COOH); ^13^C NMR (CDCl_3_; *δ*, ppm): *E*: 23.6 (CH_3_); 28.4 (CH); 49.7 (CH_2_); 124.4 (C-4′); 128.5 (C-6′); 130.0; 130.6 (C-3′; C-5′); 137.8 (C-2′); 147.7 (C-1′); 170.9 (CO); *Z*: 52.3 (CH_3_); 34.4 (C-2′); 148.8 (C-1′); 173.4 (CO).

#### *N*-Nitroso-*N*-(4,6-dibromo-2-isopropylphenyl)glycine (2c)

The same procedure as for 2a white crystals were crystallized from cyclohexane; yield 78%; mp 170–172 °C (desc.); anal. for C_11_H_12_Br_2_N_2_O_3_ (380.03): found: C 35.12; H 3.50; Br 42.44; N 7.69. Calcd: C 34.76; H 3.18; Br 42.05; N 7.37; ^1^H NMR (CDCl_3_; *δ*, ppm; *J*, Hz): *E*: 1.18; 1.21 (2d, 6H, 6.7; CH_3_); 3.38 (sep., 1H, 6.7; C**H**Me_2_); 3.95; 4.94 (2d, 2H, 16.8; CH_2_); 7.59 (d, 1H, 2.1; H-3′); 7.74 (d, 1H, 2.1; H-5′); *Z*: 1.10 (d, 6H, 6.7; CH_3_); 2.82 (sep., 1H, 6.7; C**H**Me_2_); 4.91; 5.78 (2d, 2H, 17.8; CH_2_); 7.46 (d, 1H, 2.1; H-3′); 7.65 (dd, 1H, 2.1; 2.2; H-5′); ^13^C NMR (CDCl_3_; *δ*, ppm): *E*: 23.1; 24.1 (CH_3_); 28.9 (CH); 51.2 (CH_2_); 122.2 (C-6′); 125.8 (C-4′); 130.4 (C-3′); 133.8 (C-5′); 136.3 (C-2′); 151.8 (C-1′); 171.2 (CO); *Z*: 23.4; 23.6 (CH_3_); 29.8 (CH); 54.2 (CH_2_); 130.1 (C-3′); 134.0 (C-5′); 173.4 (CO). ^13^C NMR (CDCl_3_ + TFA; *δ*, ppm): *E*: 23.4; 24.5 (CH_3_); 28.8 (CH); 49.8 (CH_2_); 122.8 (C-6′); 124.9 (C-4′); 130.1 (C-3′); 133.5 (C-5′); 136.9 (C-2′); 152.2 (C-1′); 171.2 (CO); *Z*: 23.8; 23.9 (CH_3_); 29.7 (CH); 53.6 (CH_2_); 129.7 (C-3′); 133.6 (C-5′); 173.6 (CO).

### General procedure for the synthesis of 3-(2-isopropylphenyl)sydnones 3–5

*N*-nitroso-*N*-(2-isopropylphenyl)glycine 20 mmol was dissolved in 30 mL acetic anhydride. On this solution is added 1 mL of dried pyridine. The reaction mixture is gently heated on a water bath at reduced pressure in order to evaporate the excess acetic anhydride and the acetic acid resulted from the reaction. The sydnone precipitates as oil which crystallizes upon cooling.

#### 3-(2-Isopropylphenyl)sydnone (3a)

White crystals were crystallized from ethanol; yield 87%; mp 68–69 °C. Anal. for C_11_H_12_N_2_O_2_ (204.22): found: C 65.02; H 6.31; N 14.02. Calcd: C 64.69; H 5.92; N 13.71; ^1^H NMR (CDCl_3_; *δ*, ppm; *J*, Hz): 1.25 (d, 6H, 6.9; CH_3_); 2.84 (sep., 1H, 6.9; CHMe_2_); 6.49 (s, 1H, H-4); 7.37 (dd, 1H, 7.8; 1.6; H-3′); 7.39–7.45 (m, 1H, H-5′); 7.57 (dd, 1H, 7.8; 1.6; H-6′); 7.62–7.67 (m, 1H, H-4′); ^1^H NMR (DMSO-*d*_6_; *δ*, ppm; *J*, Hz): 1.20 (d, 6H, 6.9; CH_3_); 2.74 (sep., 1H, 6.9; CHMe_2_); 7.46–7.56 (m, 2H, H-3′, H-5′); 7.55 (s, 1H, H-4); 7.63–7.75 (m, 2H, H-4′, H-6′); ^13^C NMR (CDCl_3_; *δ*, ppm): 24.0 (CH_3_); 28.0 (CHMe_2_); 97.8 (C-4); 125.2 (C-3′); 127.0 (C-5′); 127.5 (C-6′); 132.7 (C-1′); 144.1 (C-2′); 168.8 (CO).

#### 3-(4-Bromo-2-isopropylphenyl)sydnone (3b)

White crystals were crystallized from ethanol; yield 72%; mp 100–101 °C. Anal. for C_11_H_11_BrN_2_O_2_ (283.12): found: C 46.94; H 4.31; Br 28.60; N 10.22. Calcd: C 46.66; H 3.91; Br 28.22; N 9.89; ^1^H NMR (CDCl_3_, *δ*, ppm, *J*, Hz): 1.26 (d, 6H, 6.9; Me); 2.83 (sep., 1H, 6.9; C**H**Me); 6.47 (s, 1H, H-4); 7.27 (d, 1H, 8.4; H-6′); 7.56 (dd, 1H, 8.4; 2.1; H-5′); 7.68 (d, 1H, 2.1; H-3′); ^13^C NMR (CDCl_3_, *δ*, ppm): 23.8 (Me); 28.2 (**C**HMe); 97.6 (C-4); 126.8 (C-6′); 127.1 (C-4′); 130.4 (C-5′); 131.0 (C-3′); 131.6 (C-2′); 146.3 (C-1′); 168.4 (CO). ^1^H NMR (DMSO-*d*_6_, *δ*, ppm, *J*, Hz): 1.19 (d, 6H, 6.8; Me); 2.71 (sep., 1H, 6.8; C**H**Me); 7.53 (s, 1H, H-4); 7.64 (d, 1H, 8.4; H-6′); 7.71 (dd, 1H, 8.4; 2.1; H-5′); 7.89 (d, 1H, 2.1; H-3′). ^13^C NMR (DMSO-*d*_6_; *δ*, ppm): 23.3 (Me); 28.2 (**C**HMe); 99.3 (C-4); 126.2 (C-6′); 128.1 (C-4′); 130.3, 130.4 (C-3′, C-5′); 131.7 (C-2′); 146.2 (C-1′); 168.2 (CO).

#### 3-(4,6-Dibromo-2-isopropylphenyl)sydnone (3c)

White crystals were crystallized from ethanol; yield 54%; mp 169–171 °C. Anal. for C_11_H_10_Br_2_N_2_O_2_ (362.10): found: C 36.77; H 3.04; Br 44.46; N 8.09. Calcd: C 36.49; H 2.78; Br 44.14; N 7.74; ^1^H NMR (CDCl_3_; *δ*, ppm; *J*, Hz): 1.24; 1.25 (2d, 6H, 6.9; 2CH_3_); 2.63 (sep., 1H, 6.9; **C**HMe_2_); 6.47 (s, 1H, H-4); 7.63 (d, 1H, 2.0; H-3′); 7.80 (d, 1H, 2.0; H-5′); ^13^C NMR (CDCl_3_; *δ*, ppm): 23.6; 24.2 (2CH_3_); 29.4 (**C**HMe_2_); 98.1 (C-4); 120.3 (C-6′); 127.3 (C-4′); 129.7 (C-3′); 130.9 (C-1′); 133.8 (C-5′); 148.9 (C-1′); 168.5 (CO).

### General procedure for obtaining 4-brom-3-(2-isopropylphenyl)sydnones (4a,b)

3-(2-Isopropylphenyl)sydnone 10 mmol and 12 mmol sodium acetate anhydrous are dissolved in 15 mL glacial acetic acid. Over the reaction mixture are added under stirring and on an ice bath 11 mmol Br_2_ dissolved in 15 mL glacial acetic acid. The stirring is kept 10–15 minutes at room temperature and then the reaction mixture is poured over 150 mL of cold water. The formed precipitate is removed by filtration and washed with water on the filter paper.

#### 4-Bromo-3-(2-isopropylphenyl)sydnone (4a)

White crystals were crystallized from isopropanol; yield 85%; mp 91–92 °C. Anal. for C_11_H_11_BrN_2_O_2_ (283.12): found: C 46.95; H 4.22; Br 28.61; N 10.9; calcd: C 46.66; H 3.91; Br 28.22; N 9.89. ^1^H NMR (CDCl3, *δ*, ppm, *J*, Hz): 1.25 (d, 6H, 6.8; Me); 2.64 (sep., 1H, 6.8; CHMe2); 7.29 (dd, 1H, 7.9; 1.5; H-3′); 7.42–7.49 (m, 1H, H-5′); 7.59 (dd, 1H, 7.9; 1.5; H-6′); 7.65–7.71 (m, 1H, H-4′); ^13^C NMR (CDCl3, *δ*, ppm): 23.3 (bs, Me); 28.4 (CHMe2); 86.1 (C-4); 126.1; 127.3; 127.7 (C-3′, C-5′, C-6′); 131.6 (C-1′); 145.0 (C-2′); 165.5 (CO).

#### 4-Bromo-3-(4-bromo-2-isopropylphenyl)sydnone (4b)

White crystals were crystallized from ethanol; yield 82%. mp 142–144 °C. Anal. for C_11_H_10_Br_2_N_2_O_2_ (362.10): found: C 36.77; H 3.02; Br 44.53; N 8.02. Calcd: C 36.49; H 2.78; Br 44.14; N 7.74; ^1^H NMR (CDCl_3_, *δ*, ppm; *J*, Hz): 1.24 (d, 6H, 6.8; Me); 2.61 (sep., 1H, 6.8; C**H**Me); 7.19 (d, 1H, 8.5; H-6′); 7.59 (dd, 1H, 8.5; 2.2; H-5′); 7.71 (d, 1H, 2.2; H-3′); ^13^C NMR (CDCl_3_, *δ*, ppm): 23.6 (sl, Me); 28.5 (**C**HMe); 86.1 (C-4); 127.6 (C-6′); 127.7 (C-4′); 130.4 (C-3′); 130.7 (C-5′); 131.1 (C-2′); 147.1 (C-1′); 165.1 (CO).

#### 4-Iodo-3-(2-isopropylphenyl)sydnone (5a)

3-(2-Isopropylphenyl)sydnone 10 mmol and 15 mmol anhydrous sodium acetate are dissolved in 15 mL acid acetic glacial. A solution of 12 mmol ICl in 5 mL acetic acid glacial is added under stirring and then the reaction mixture is stirred for 10 min at room temperature and another 1 h at 50–60 °C. After this, the reaction mixture is poured over 150 mL of cold water. The precipitated 4-iodosydnone is filtered and washed with water triturated with ethylic ether and crystalized from ethanol as white crystals. Yield 82%; mp 179–180 °C. Anal. for C_11_H_11_IN_2_O_2_ (330.12): found: C 40.38; H 3.67; I 38.83; N 8.79. Calcd: C 40.02; H 3.35; I 38.44; N 8.48; ^1^H NMR (CDCl_3_, *δ*, ppm, *J*, Hz): 1.21; 1.22 (2d, 6H, Me); 2.55 (sep., 1H, 6.8; C**H**Me_2_); 7.23 (dd, 1H, 8.0; 1.5; H-3′); 7.39–7.45 (m, 1H, H-5′); 7.56 (dd, 1H, 8.0; 1.5; H-6′); 7.62–7.68 (m, 1H, H-4′); ^13^C NMR (CDCl_3_, *δ*, ppm): 22.7; 24.3 (2s, Me); 53.5 (C-4); 126.2; 127.6; 127.7 (C-3′, C-5′, C-6′); 132.9 (C-4′); 133.2 (C-1′); 144.8 (C-2′); 168.6 (CO).

#### 4-Iodo-3-(4-bromo-2-isopropylphenyl)sydnone (5b)

White crystals were crystallized from isopropanol; yield 82%; mp 195–197 °C. Anal. for C_11_H_10_BrIN_2_O_2_ (409.02): N 7.04. Calcd: N 6.85; ^1^H NMR (CDCl_3_; *δ*, ppm; *J*, Hz): 1.24 (bd 6H, 6.9; Me); 2.56 (sep., 1H, 6.9; C**H**Me_2_); 7.15 (d, 1H, 8.4; H-6′); 7.59 (dd, 1H, 8.4; 2.1; H-5′); 7.70 (d, 1H, 2.1; H-3′); ^13^C NMR (CDCl_3_; *δ*, ppm): 22.7; 24.4 (2s, Me); 28.5 (**C**HMe_2_); 53.1 (C-4); 127.5 (C-4′); 127.7 (C-6′); 130.6 (C-3′); 131.1 (C-5′); 131.9 (C-2′); 146.9 (C-1′); 168.3 (CO).

### General procedure for the synthesis of the 1-arylpyrazoles 6

#### Dimethyl 1-(2-isopropylphenyl)pyrazole-3,4-dicarboxylate (6a)

10 mmol sydnone 4a and 12 mmol DMAD are heated under reflux in 20 mL toluene for 8 h. The solvent is evaporated and the resulted oil is eluted on an Al_2_O_3_ column using CH_2_Cl_2_ as solvent. The pyrazole 6a is crystallized from methanol as white crystals. Yield 87%; mp 62–63 °C; anal. for C_16_H_18_N_2_O_4_ (302.3): found: C 63.87; H 6.33; N 9.55. Calcd: C 63.56; H 6.00; N 9.27. ^1^H NMR (CDCl_3_; *δ*, ppm; *J*, Hz): 1.18 (d, 6H, 6.8; CH_3_); 2.80 (sep., 1H, 6.8; C**H**Me_2_); 3.89; 3.98 (2s, 6H, OCH_3_); 7.6–7.30 (m, 2H, H-3′; H-5′); 7.44–7.49 (m, 2H, H-4′; H-6′); ^13^C NMR (CDCl_3_; *δ*, ppm): 23.7 (CH_3_); 27.8 (C**H**Me_2_); 51.9; 52.5 (OCH_3_); 115.4 (C-4); 126.4; 126.7; 126.8 (C-3′; C-4′; C-5′); 130.3 (C-6′); 136.0 (C-5); 137.3 (C-1′); 143.9 (C-3); 144.8 (C-2′); 162.0 (2CO).

## Conflicts of interest

There are no conflicts to declare.

## Supplementary Material

RA-010-D0RA02368J-s001

RA-010-D0RA02368J-s002
