# Supplementary material for: Introducing chirality in halogenated 3-arylsydnones and their corresponding 1-arylpyrazoles obtained by 1,3-dipolar cycloaddition
Source: RSC Adv. 2020 Apr 21;10(27):15656–64. doi: 10.1039/d0ra02368j (PMC9052417; doi:10.1039/d0ra02368j)
Supplement: RA-010-D0RA02368J-s001 [file RA-010-D0RA02368J-s001.pdf]

Electronic Supplementary Material (ESI) for RSC Advances.
This journal is © The Royal Society of Chemistry 2020

Supplementary Information

Introducing chirality in halogenated 3-arylsydnones and corresponding 1-arylpyrazoles obtained by 1,3-dipolar cycloaddition

Marcel Mirel Popa[a], Sergiu Shova[b], Madalina Hrubaru[a], Loredana Barbu[a], Constantin Draghici[a], Florea Dumitrascu*[a], Denisa Dumitrescu[c]

Address: [a]Center of Organic Chemistry ''C. D. Nenitzescu'', Roumanian Academy, Spl Independentei 202B, 060023 Bucharest, Romania; [b]"Petru Poni" Institute of Macromolecular Chemistry, Aleea Gr. Ghica Voda 41A, Iasi 700487, Romania [c]Romania Ovidius University Constanta, Faculty of Pharmacy, Str. Cpt. Av. Al. Serbanescu 6, Campus Corp C, Constanta 900470, Romania

Contents:

1. General..2
2. Synthesis and NMR characterization.......................................2
3. X-ray crystalography..10

1. General

Melting points were measured using a Boetius hot plate microscope and are uncorrected. ^{1}H-NMR and ^{13}C-NMR spectra were recorded on a Varian Gemini 300BB operating at 300 MHz for ^{1}H and 75 MHz for ^{13}C. The spectra were recorded in $CDCl_3$ or DMSO-d_6 at 298K and the chemical shifts are relative to TMS used as the internal standard. Elemental analysis was performed on a Costech Instruments EAS 32 apparatus and the results were in agreement with the calculated values. All starting materials and solvents were purchased from common commercial suppliers and were used without further purification.

2. Synthesis and NMR characterization

The synthesis of ***N*-(2-isopropyl)phenylglycine (1a)**. M.p. 139-141°C. Yield 46%. 30 ml (0.21 mol) of 2-isopropylaniline are mixed with 17 g (0.18 mol) of monochloroaectic acid in 200 mL water and 10 mL ethanol. The reaction mixture is kept under reflux for 3 hrs. The reaction mass is cooled with water and then stirred for 1 hr on an ice bath. The formed precipitate is removed by filtration and then washed with water and with benzene after drying to remove any unreacted 2-isoporpylaniline. Anal. for $C_{11}H_{15}NO_2$ (193.2): Found: C 68.67; H 8.04; N 7.52. Calcd.: C 68.37; H 7.82; N 7.25. ^{1}H RMN ($CDCl_3$; δ, ppm; *J*, Hz): 1.28 (d, 6H, 6.8; Me); 2.96 (sep., 1H, 6.8; C**H**Me_2); 4.03 (s, 2H, CH_2); 6.15 (sl, 2H, COOH, NH); 6.54 (dd, 1H, 7.6; 1.5; H-6'); 6.83 (td, 7.6; 1.7; H-4'); 7.13 (td, 7.6; 1.5; H-5'); 7.19 (dd, 7.6; 1.7; H-3'). ^{13}C RMN($CDCl_3$; δ, ppm): 22.3 (Me); 27.3 (**C**HMe_2); 46.0 (CH_2); 110.8 (C-6'); 118.9 (C-4'); 125.3 (C-3'); 126.8 (C-5'); 133.0 (C-2'); 143.3 (C-1'); 176.1 (CO).

2.1 The synthesis of *N*-phenylglycines 1b,c.

N-(2-Isopropyl)phenylglycine 3.9 g (20 mmol) are suspended in 20 mL glacial acetic acid. Over this solution is added by stirring 20 mmol Br_2 for **1b** or 40 mmol Br_2 for **1c** dissolved in 5 mL acetic acid. The reaction is kept under stirring for ca. 15-20 min and then the reaction mass is poured over cold water under continuous stirring. The formed precipitate is filtered and washed with water on the filter paper, and then the compound is dried.

***N*-(4-Bromo-2-isopropylphenyl)glycine (1b)**. White powder crystallized from ethyl acetate. Yield 94 %, m.p. 191-193 ^{o}C. Anal. for $C_{11}H_{14}BrNO_2$ (272.14): Found: C 48.87; H 5.47; Br 29.76; N 5.42. Calcd.: C 48.55; H 5.19; Br 29.36; N 5.15. ^{1}H RMN($CDCl_3$+TFA; δ, ppm; *J*, Hz): 1.23 (d, 6H, 6.8; $2CH_3$); 3.25 (sep., 1H, 6.8; C**H**Me_2); 3.91 (s, 2H, CH_2); 7.33 (d, 1H, 8.6; H-6'); 7.51 (dd, 1H, 8.6; 2.2; H-5'); 7,64 (d, 1H, 2.2; H-3'); ^{13}C RMN($CDCl_3$+TFA; δ, ppm): 23.6 (CH_3); 27.8 (**C**HMe_2); 52.5 (CH_2): 124.2 (C-6'); 124.9 (C-4'); 130.8 (C-2'); 131.3; 131.6 (C-3'; C-5'); 143.7 (C-1'); 168.9 (CO).

***N*-(4,6-Dibromo-2-isopropylphenil)glycine(1c)**. White crystals were crystallized from cyclohexane; Yield 76%; m.p. 103-105°C (desc.). Anal. for $C_{11}H_{13}Br_2NO_2$ (351.04): Found: C 37.97; H 4.06; Br 45.90; N 4.31. Calcd.: C 37.63 ; H 3.73; Br 45.52; N 3.99. ^{1}H RMN ($CDCl_3$; δ, ppm; *J*, Hz): 1.23 (d, 6H; CH_3); 3.25 (sep., 1H, 6.8; C**H**Me_2); 3.91 (s, 2H, CH_2); 7.29 (d, 1H, 2.2; H-3'); 7.53 (d, 1H, 2.2; H-5'); 7.70 (sl, 2H, N**H**, COO**H**). ^{13}C RMN($CDCl_3$; δ, ppm): 23.7 ($2CH_3$); 28.7 (CHMe_2); 50.3 (CH_2); 116.7 (C-6'); 119.2 (C-4'); 129.0 (C-3'); 142.0 (C-1); 144.9 (C-1'); 176.7 (CO).

2.2 Procedures for obtaining *N*-nitroso-*N*-phenylglycines 2a-c

N-(2-Isopropylphenyl)glycine 20 mmol are dissolved in 40 mL solution of 5% NaOH and 21.5 mmol of $NaNO_2$ are added. Over this mixture 10 mL conc. HCl are added under vigorous stirring and coling on an ice bath, until the pH will reach a value of 1.5-2. The stirring continues for another 15 min while the nitrosoderivative precipitates. The N-nitrosophenylglicine is filtered washed with water on the filter and let to dry in air.

***N*-Nitroso-*N*-(2-isopropylphenyl)glycine (2a).** White crystals obtained from ethanol-benzene mixture; Yield 94%; m.p. 104-106°C (desc.). Anal.: $C_{11}H_{14}N_2O_3$ (222.24): Found: C 59.77; H 6.66; N 12.42. Calcd: C 59.45; H 6.35; N 12.21. ^{1}H RMN ($CDCl_3$; δ, ppm; *J*, Hz): *E*(95%): 1.23 (d, 6H, 6.9; CH_3); 2.94 (sep., 1H, 6.9; **CH**Me_2); 4.52 (s, 2H, CH_2); 7.31-7.44 (m, 2H, H-3; H-5); 7.49-7.53 (m, 2H, H-4; H-6); *Z* (5%): 1.12; 1.17 (2d, 6H, 6.8; CH_3); 2.53 (sep., 1H, 6.8; **CH**Me_2); 4.99; 5.55 (2d, 2H, 17.8; CH_2); ^{13}C RMN($CDCl_3$; δ, ppm): *E* (95%): 24.0 (CH_3); 28.2 (**C**HMe_2); 50.1 (CH_2); 126.9; 127.3; 130.3 (C-3', C-4', C-5', C-6'); 138.9 (C-1'); 145.4 (C-2'); 171.1 (CO). *Z* (5%): 23.7; 23.9 (CH_3); 28.6 (**C**HMe_2); 54.9 (CH_2); CO?.

***N*-Nitroso-*N*-(4-bromo-2-isopropylphenyl)glycine (2b).** The same procedure as for **1a** is employed. The compound precipitates as oil which is extracted twice with 15 mL CH_2Cl_2. This solution is dried over $CaCl_2$ and after the evaporation of CH_2Cl_2 the amorphous mass is crystalized from ethyl acetate-benzene. Yield 89%; m.p. 121-123 ^oC (desc.). Anal. for $C_{11}H_{13}BrN_2O_3$ (301.14): Found: C 44.09; H 4.72; Br 26.94 N 9.59. Calcd.: C 43.87; H 4.35; Br 26.53; N 9.30; ^1H RMN ($CDCl_3$; δ, ppm; *J*, Hz): *E*: 1.22 (d, 6H, 6.9; CH_3); 2.90 (sep., 1H, 6.9; $CHMe_2$); 4.49 (s, 2H, CH_2); 7.28 (d, 1H, 8.4; H-6'); 7.47 (dd, 1H, 8.4; 2.2; H-5'); 7.61 (d, 1H, 2.2; H-3'); 9.45 (sl, 2H, COOH); *Z*: 1.10; 1.16 (d, 6H, 6.9; CH_3); 2.48 (sep., 1H, 6.9; C**H**Me_2); 4.94; 5.53 (2d, 2H, 17.8; CH_2); 6.95 (d, 1H, 8.4; H-6'); 7.37 (dd, 1H, 8.4; 2.2; H-5'); 7.50 (d, 1H, 2.2; H-3'); 9.45 (bs, 2H, COOH).

^{13}C RMN($CDCl_3$; δ, ppm): *E*: 23.6 (CH_3); 28.4 (CH); 49.7 (CH_2); 124.4 (C-4'); 128.5 (C-6'); 130.0; 130.6 (C-3'; C-5'); 137.8 (C-2'); 147.7 (C-1'); 170.9 (CO); *Z*: 52.3 (CH_3); 34.4 (C-2'); 148.8 (C-1'); 173.4 (CO).

***N*-Nitroso-*N*-(4,6-dibromo-2-isopropylphenil)glycine (2c**). The same procedure as for **2b**. White crystals were crystallized from cyclohexane; Yiled 78%; m.p. 170-2^oC (desc.); Anal. for $C_{11}H_{12}Br_2N_2O_3$ (380.03): Found: C 35.12; H 3.50; Br 42.44; N 7.69. Calcd.: C 34.76; H 3.18; Br 42.05; N 7.37; ^1H RMN ($CDCl_3$; δ, ppm; *J*, Hz): *E*: 1.18; 1.21 (2d, 6H, 6.7; CH_3); 3.38 (sep., 1H, 6.7; C**H**Me_2); 3.95; 4.94 (2d, 2H, 16.8; CH_2); 7.59 (d, 1H, 2.1; H-3'); 7.74 (d, 1H, 2.1; H-5'); *Z*: 1.10 (d, 6H, 6.7; CH_3); 2.82 (sep., 1H, 6.7; C**H**Me_2); 4.91; 5.78 (2d, 2H, 17.8; CH_2); 7.46 (d, 1H, 2.1; H-3'); 7.65 (dd, 1H, 2.1; 2.2; H-5'); ^{13}C RMN ($CDCl_3$; δ, ppm): *E*: 23.1; 24.1 (CH_3); 28.9 (CH); 51.2 (CH_2); 122.2 (C-6'); 125.8 (C-4'); 130.4 (C-3'); 133.8 (C-5'); 136.3 (C-2'); 151.8 (C-1'); 171.2 (CO); *Z*: 23.4; 23.6 (CH_3); 29.8 (CH); 54.2 (CH_2); 130.1 (C-3'); 134.0 (C-5'); 173.4 (CO). ^{13}C RMN($CDCl_3$ + TFA; δ, ppm): *E*: 23.4; 24.5 (CH_3); 28.8 (CH); 49.8 (CH_2); 122.8 (C-6'); 124.9 (C-4'); 130.1 (C-3'); 133.5 (C-5'); 136.9 (C-2'); 152.2 (C-1'); 171.2 (CO); *Z*: 23.8; 23.9 (CH_3); 29.7 (CH); 53.6 (CH_2); 129.7 (C-3'); 133.6 (C-5'); 173.6 (CO).

2.3 General procedure for the synthesis of 3-(2-isopropylphenyl)sydnones 3a-c and 4a,b

N-nitroso-*N*-(2-izopropylphenyl)glycine 20 mmol was dissolved in 30 mL acetic anhydride. On this solution is added 1 mL of dried pyridine. The reaction mixture is gently heated on a water bath at reduced pressure in order to evaporate the excess acetic anhydride and the acetic acid resulted from the reaction. The sydnone precipitates as oil which crystallizes upon cooling.

3-(2-Isopropylphenyl)sydnone (3a). White crystals were crystallized from ethanol; Yield 87%; m.p. 68-69 ^{o}C. Anal. for $C_{11}H_{12}N_2O_2$ (204.22): Found: C 65.02; H 6.31; N 14.02. Calcd.: C 64.69; H 5.92; N 13.71; ^{1}H RMN (CDCl$_3$; δ, ppm; *J*, Hz): 1.25 (d, 6H, 6.9; CH$_3$); 2.84 (sep., 1H, 6.9; CHMe$_2$); 6.49 (s, 1H, H-4); 7.37 (dd, 1H, 7.8; 1.6; H-3'); 7.39-7.45 (m, 1H, H-5'); 7.57 (dd, 1H, 7.8; 1.6; H-6'); 7.62-7.67 (m, 1H, H-4'); ^{1}H RMN (DMSO-d$_6$; δ, ppm; *J*, Hz): 1.20 (d, 6H, 6.9; CH$_3$); 2.74 (sep., 1H, 6.9; CHMe$_2$); 7.46-7.56 (m, 2H, H-3', H-5'); 7.55 (s, 1H, H-4); 7.63-7.75 (m, 2H, H-4', H-6'); ^{13}C RMN (CDCl$_3$; δ, ppm): 24.0 (CH$_3$); 28.0 (CHMe$_2$); 97.8 (C-4); 125.2 (C-3'); 127.0 (C-5'); 127.5 (C-6'); 132.7 (C-1'); 144.1 (C-2'); 168.8 (CO).

3-(4-Bromo-2-isopropylphenyl)sydnone (**3b**). White crystals were crystallized from ethanol; Yield 72%; m.p. 100-101°C. Anal. for $C_{11}H_{11}BrN_2O_2$ (283.12): Found: C 46.94; H 4.31; Br 28.60; N 10.22. Calcd.: C 46.66; H 3.91; Br 28.22; N 9.89; ^{1}H RMN(CDCl$_3$, δ, ppm, *J*, Hz): 1.26 (d, 6H, 6.9; Me); 2.83 (sep., 1H, 6.9; C**H**Me); 6.47 (s, 1H, H-4); 7.27 (d, 1H, 8.4; H-6'); 7.56 (dd, 1H, 8.4; 2.1; H-5'); 7.68 (d, 1H, 2.1; H-3'); ^{13}C RMN (CDCl$_3$, δ, ppm;): 23.8 (Me); 28.2 (**C**HMe); 97.6 (C-4); 126.8 (C-6'); 127.1 (C-4'); 130.4 (C-5'); 131.0 (C-3'); 131.6 (C-2'); 146.3 (C-1'); 168.4(CO).

^{1}H RMN (DMSO-d$_6$, δ, ppm, *J*, Hz): 1.19 (d, 6H, 6.8; Me); 2.71 (sep., 1H, 6.8; C**H**Me); 7.53 (s, 1H, H-4); 7.64 (d, 1H, 8.4; H-6'); 7.71 (dd, 1H, 8.4; 2.1; H-5'); 7.89 (d, 1H, 2.1; H-3').

^{13}C RMN (DMSO-d$_6$; δ, ppm): 23.3 (Me); 28.2 (**C**HMe); 99.3 (C-4); 126.2 (C-6'); 128.1 (C-4'); 130.3 130.4 (C-3', C-5'); 131.7 (C-2'); 146.2 (C-1'); 168.2 (CO).

3-(4,6-Dibromo-2-isopropylphenyl)sydnone (**3c**). White crystals were crystallized from ethanol; Yield 54%; m.p. 169-171°C. Anal. for $C_{11}H_{10}Br_2N_2O_2$ (362.10): Found: C 36.77; H 3.04; Br 44.46 N 8.09. Calcd.: C 36.49; H 2.78; Br 44.14; N 7.74; ^{1}H RMN (CDCl$_3$; δ, ppm; *J*, Hz): 1.24; 1.25 (2d, 6H, 6,9; 2CH$_3$); 2.63 (sep., 1H, 6.9; **C**HMe$_2$); 6.47 (s, 1H, H-4); 7.63 (d, 1H, 2.0; H-3'); 7.80 (d, 1H, 2.0; H-5'); ^{13}C RMN(CDCl$_3$; δ, ppm): 23.6; 24.2

$(2CH_3)$; 29.4 (**C**HMe_2); 98.1 (C-4); 120.3 (C-6'); 127.3 (C-4'); 129.7 (C-3'); 130.9 (C-1'); 133.8 (C-5'); 148.9 (C-1'); 168.5 (CO).

2.4 General procedure for obtaining 4-bromo-3-(2-isopropylphenyl)sydnones 4a,b. 3-(2-Isopropylphenyl)sydnone 10 mmol and 12 mmol sodium acetate anhydrous are dissolved in 15 mL glacial acetic acid. Over the reaction mixture are added under stirring and on an ice bath 11 mmol Br_2 dissolved in 15 mL glacial acetic acid. The stirring is kept 10-15 minutes at room temperature and then the reaction mixture is poured over 150 mL of cold water. The formed precipitate is removed by filtration and washed with water on the filter paper.

4-Bromo-3-(2-isopropylphenyl)sydnone (**4a**). White crystals were crystallized from isopropanol; Yield 85%; m.p. 91-92 ^oC. Anal. for $C_{11}H_{11}BrN_2O_2$ (283.12): Found: C 46.95; H 4.22; Br 28.61; N 10.9; Calcd.: C 46.66; H 3.91; Br 28.22; N 9.89. ^1H RMN(CDCl3, δ, ppm, *J*, Hz): 1.25 (d, 6H, 6.8; Me); 2.64 (sep., 1H, 6.8; CHMe2); 7.29 (dd, 1H, 7.9; 1.5; H-3'); 7.42-7.49 (m, 1H, H-5'); 7.59 (dd, 1H, 7.9; 1.5; H-6'); 7.65-7.71 (m, 1H, H-4'); ^{13}C RMN(CDCl3, δ, ppm): 23.3 (bs, Me); 28.4 (CHMe2); 86.1 (C-4); 126.1; 127.3; 127.7 (C-3', C-5', C-6'); 131.6 (C-1'); 145.0 (C-2'); 165.5 (CO).

4-Bromo-3-(4-bromo-2-isopropylphenyl)sydnone (**4b**). White crystals were crystallized from ethanol; Yield 82%. m.p. 142-144 ^oC. Anal. for $C_{11}H_{10}Br_2N_2O_2$ (362.10): Found: C 36.77; H 3.02; Br 44.53; N 8.02. Calcd.: C 36.49; H 2.78; Br 44.14; N 7.74; ^1H RMN ($CDCl_3$, δ, ppm; *J*, Hz): 1.24 (d, 6H, 6.8; Me); 2.61 (sep., 1H, 6.8; C**H**Me); 7.19 (d, 1H, 8.5; H-6'); 7.59 (dd, 1H, 8.5; 2.2; H-5'); 7.71 (d, 1H, 2.2; H-3'); ^{13}C RMN ($CDCl_3$, δ, ppm): 23.6 (sl, Me); 28.5 (**C**HMe); 86.1 (C-4); 127.6 (C-6'); 127.7 (C-4'); 130.4 (C-3'); 130.7 (C-5'); 131.1 (C-2'); 147.1 (C-1'); 165.1 (CO).

4-Iodo-3-(2-isopropylphenyl)sydnone (**5a**). 3-(2-isopropylphenyl)sydnone 10 mmol and 15 mmol anhydrous sodium acetate are dissolved in 15 mL acid acetic glacial. A solution of 12 mmol ICl in 5 mL acetic acid glacial is added under stirring and then the reaction mixture is stirred for 10 min at room temperature and another 1 hr at 50-60 ^oC. After this, the reaction mixture is poured over 150 mL of cold water. The precipitated 4-iodosydnone is filtered and washed with water titurated with ethylic ether and crystalized from ethanol as white crystals. Yield 82%; m.p. 179-180^0C. Anal. for $C_{11}H_{11}IN_2O_2$ (330.12): Found: C 40.38; H 3.67; I 38.83; N 8.79. Calcd.: C 40.02; H 3.35;

I 38.44; N 8.48; ^{1}H RMN ($CDCl_3$, δ, ppm, *J*, Hz): 1.21; 1.22 (2d, 6H, Me); 2.55 (sep., 1H, 6.8; C**H**Me_2); 7.23 (dd, 1H, 8.0; 1.5; H-3'); 7.39-7.45 (m, 1H, H-5'); 7.56 (dd, 1H, 8.0; 1.5; H-6'); 7.62-7.68 (m, 1H, H-4'); ^{13}C RMN ($CDCl_3$, δ, ppm): 22.7; 24.3 (2s, Me); 53.5 (C-4); 126.2; 127.6; 127.7 (C-3', C-5', C-6'); 132.9 (C-4'); 133.2 (C-1'); 144.8 (C-2'); 168.6 (CO).

4-Iodo-3-(4-bromo-2-isopropylphenyl)sydnone (**5b**). White crystals were crystallized from isopropanol; Yield 82%; m.p. 195-197 °C. Anal. for $C_{11}H_{10}BrIN_2O_2$ (409.02): N 7.04. Calcd.N 6.85; ^{1}H RMN ($CDCl_3$; δ, ppm; *J*, Hz): 1.24 (dl, 6H, 6.9; Me); 2.56 (sep., 1H, 6.9; C**H**Me_2); 7.15 (d, 1H, 8.4; H-6'); 7.59 (dd, 1H, 8.4; 2.1; H-5'); 7.70 (d, 1H, 2.1; H-3'); ^{13}C RMN ($CDCl_3$; δ, ppm): 22.7; 24.4 (2s, Me); 28.5 (**C**HMe_2); 53.1 (C-4); 127.5 (C-4'); 127.7 (C-6'); 130.6 (C-3'); 131.1 (C-5'); 131.9 (C-2'); 146.9 (C-1'); 168.3 (CO).

2.5 General procedure for the synthesis of the 1-arylpyrazoles 6a-c

Dimethyl 1-(2-isopropylphenyl)pyrazole-3,4-dicarboxylate (6a). 10 Mmol sydnone **4a** and 12 mmol DMAD are heated under reflux in 20 mL toluene for 8 hrs. The solvent is evaporated and the resulted oil is eluted on an Al_2O_3 column using CH_2Cl_2 as solvent. The pyrazole **6a** is crystallized from methanol as white crystals. Yield 87%; m.p. 62-63 °C; Anal. for $C_{16}H_{18}N_2O_4$ (302.3): Found: C 63.87; H 6.33; N 9.55. Calculat: C 63.56; H 6.00; N 9.27. ^{1}H RMN ($CDCl_3$; δ, ppm; *J*, Hz): 1.18 (d, 6H, 6.8; CH_3); 2.80 (sep., 1H, 6.8; C**H**Me_2); 3.89; 3.98 (2s, 6H, OCH_3); 7.6-7.30 (m, 2H, H-3'; H-5'); 7.44-7.49 (m, 2H, H-4'; H-6'); ^{13}C RMN($CDCl_3$; δ, ppm): 23.7 (CH_3); 27.8 (C**H**Me_2); 51.9; 52.5 (OCH_3); 115.4 (C-4); 126.4; 126.7; 126.8 (C-3'; C-4'; C-5'); 130.3 (C-6'); 136.0 (C-5); 137.3 (C-1'); 143.9 (C-3); 144.8 (C-2'); 162.0 (2CO).

Dimethyl 1-(4-bromo-2-isopropylphenyl)pyrazole-3,4-dicarboxylate (6b). Same procedure as for **6a**. White crystals were crystallized from isopropanol; Yield 94%; m.p. 139-140 °C. Anal. for $C_{16}H_{17}Br\ N_2O_4$ (381.22): Found: C 50.73; H 4.79; Br 21.35; N 7.59; Calcd.: C 50.41; H 4.49; Br 20.96; N 7.35; ^{1}H RMN ($CDCl_3$; δ, ppm; *J*, Hz): 1.18 (d, 6H, 6.8; CH_3); 2.79 (sep., 1H, 6.8; $CHMe_2$); 3.88; 3.98 (2s, 6H, OCH_3); 7.17 (d, 1H, 8.4; H-6'); 7.43 (dd, 1H, 8.4; 2.2; H-5'); 7.57 (d, 1H, 2.2; H-3'); 8.03 (s, 1H, H-5); ^{13}C RMN ($CDCl_3$; δ, ppm): 23.6 (CH_3); 27.9 (CH Me_2); 52.1; 52.8 (OCH_3); 115.7 (C-4); 124.4 (C-4'); 128.4; 129.6; 130.1 (C-3'; C-5'; C-6'); 135.8 (C-1'); 144.2 (C-3); 147.0 (C-2'); 161.8; 161.9 (CO).

Dimethyl 1-(4,6-dibromo-2-isopropylphenyl)pyrazole-3,4-dicarboxylate (6c). 4 Mmol of the corresponding sydnone (**3c**) and 6 mmol DMAD are heated under reflux in 20 mL xylene for 24 hrs. The solvent is evaporated under reduced pressure and the obtained oil is crystallized from ethanol as white cystals. Yield 67%; m.p. 113-115 ^oC; Anal. for $C_{16}H_{16}Br_2N_2O_4$ (460.117): Found: C 42.03; H 3.83; Br 35.11; N 6.37. Calcd.: C 41.77; H 3.50; Br 34.73; N 6.09; ^1H RMN ($CDCl_3$; δ, ppm; *J*, Hz): 1.12; 1.19 (2d, 6H, 6.9; CH_3); 2.48 (sep., 1H, 6.9; $CHMe_2$); 3.89; 3.98 (2s, 6H, 2OCH_3); 7.51 (d, 1H, 2.0; H-3'); 7.70 (d, 1H, 2.0; H-5'); 7.97 (s, 1H, H-5); 13C NMR?

Dimethyl 5-bromo-1-(2-isopropylphenyl)pyrazole-3,4-dicarboxylate (6d). Same procedure as **6c**. White crystals were crystallized from methanol; Yield 87%; m.p. 92-93°C; Anal. for $C_{16}H_{17}BrN_2O_4$ (381.2): Found: C 50.72; H 4.82; Br 21.33; N 7.59. Calcd.: C 50.41; H 4.49; Br 20.96; N 7.35; ^1H RMN ($CDCl_3$; δ, ppm; *J*, Hz): 1.17 (bd, 6H; 6.8; CH_3); 2.48 (sep., 1H, $CHMe_2$); 3.94; 3.96 (2s, 6H, OCH_3); 7.19 (dd, 1H, 7.8; 1.4; H-3'); 7.28-7.36 (m, 1H, H-5'); 7.46 (dd, 1H, 7.8; 1.8; H-4'); 7.49-7.55 (m, 1H, H-6') ^{13}C RMN ($CDCl_3$; δ, ppm): 23.1; 24.0 (CH_3); 28.2 ($CHMe_2$); 52.4; 52.8 (OCH_3); 115.5 (C-5); 120.0 (C-4); 126.5 (C-5'); 126.8 (C-6'); 128.1 (C-3'); 131.1 (C-4'); 135.7 (C-1'); 144.4 (C-3); 146.5 (C-2'); 161.5; 161.9 (CO).

Dimethyl 5-iodo-1-(2-isopropylphenyl)pyrazole-3,4-dicarboxylate (6e).[52] Same procedure as **6c**. White crystals were crystallized from isopropanol. Yield 82%; m.p. 156-157 ^oC

Dimethyl 5-bromo-1-(4-bromo-2-isopropylphenyl)pyrazole-3,4-dicarboxylate (6f). Same procedure as **6c**. White crystals were crystallized from isopropanol; Yield 86%; m.p. 103-104°C. Anal. for $C_{16}H_{16}Br_2N_2O_4$ (460.1): Found: C 42.02; H 3.79; Br 35.06; N 6.33. Calcd.: C 41.77; H 3.50; Br 34.73; N 6.09; ^1H RMN ($CDCl_3$; δ, ppm; *J*, Hz): 1.16 (d, 6H, 6.8; CH_3); 2.45 (sep., 1H, 6.8; $CHMe_2$); 3.93; 3.95 (2s, 6H, OCH_3); 7.08 (d, 1H, 8.4; H-6'); 7.45 (dd, 1H, 8.4; 2.2; H-5'); 7.58 (d, 1H, 2.2; H-3'); ^{13}C RMN($CDCl_3$; δ, ppm): 22.8; 23.8 (CH_3); 28.4 ($CHMe_2$); 52.4; 52.8 (OCH_3); 115.6 (C-5); 120.0 (C-4); 125.4 (C-4'); 129.6 (C-6'); 129.8 (C-5'); 130.3 (C-3'); 134.6 (C-1'); 144.6 (C-3); 148.7 (C-2'); 161.3; 161.6 (CO).

Dimethyl 5-iodo-1-(4-bromo2-isopropylphenyl)pyrazole-3,4-dicarboxylate (6g). Same procedure as **6c**. White crystals were crystallized from isopropanol or ethanol.

Yield 80%; m.p. 128-131 °C; Anal. for $C_{16}H_{16}BrIN_2O_4$ (507.1): Found: N 5,78. Calcd.: N 5,52; ^{1}H RMN ($CDCl_3$; δ, ppm; *J*, Hz): 1.15; 1.19 (2d, 6H, 6.8; CH_3); 2.39 (sep., 1H, 6,8; $CHMe_2$); 3.94; 3.96 (2s, 6H, OCH_3); 7.06 (d, 1H, 8.4; H-6'); 7.46 (dd, 1H, 8.4; 2.2; H-5'); 7.59 (d, 1H, 2.2; H-3'); ^{13}C RMN ($CDCl_3$; δ, ppm): 22.7; 23.9 (CH_3); 28.5 ($CHMe_2$); 52.4; 52.8 (OCH_3); 91.5 (C-5); 120.8 (C-4); 125.3 (C-4'); 129.8 (C-5'); 130.0 (C-6'); 130.3 (C-3'); 136.3 (C-1'); 145.3 (C-3); 148.7 (C-2'); 161.4; 162.1 (CO).

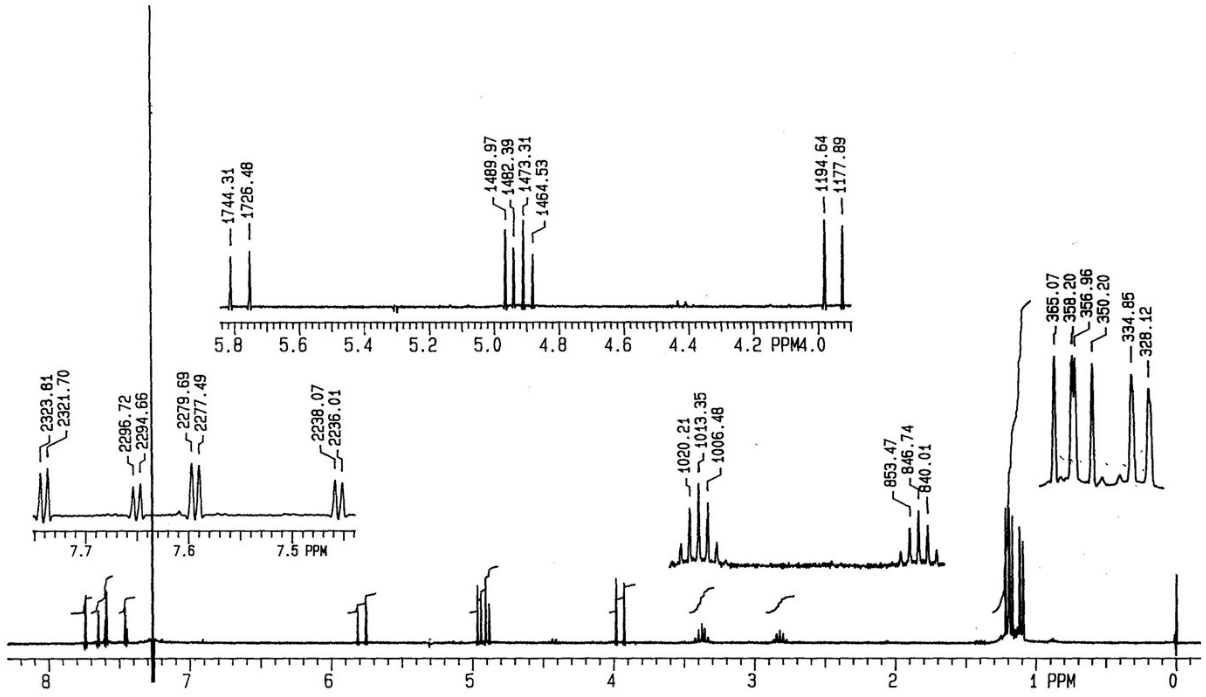

Figure 1S. ^{1}H NMR spectrum of **2c** showing the presence of both *E* and *Z* isomers in $CDCl_3$

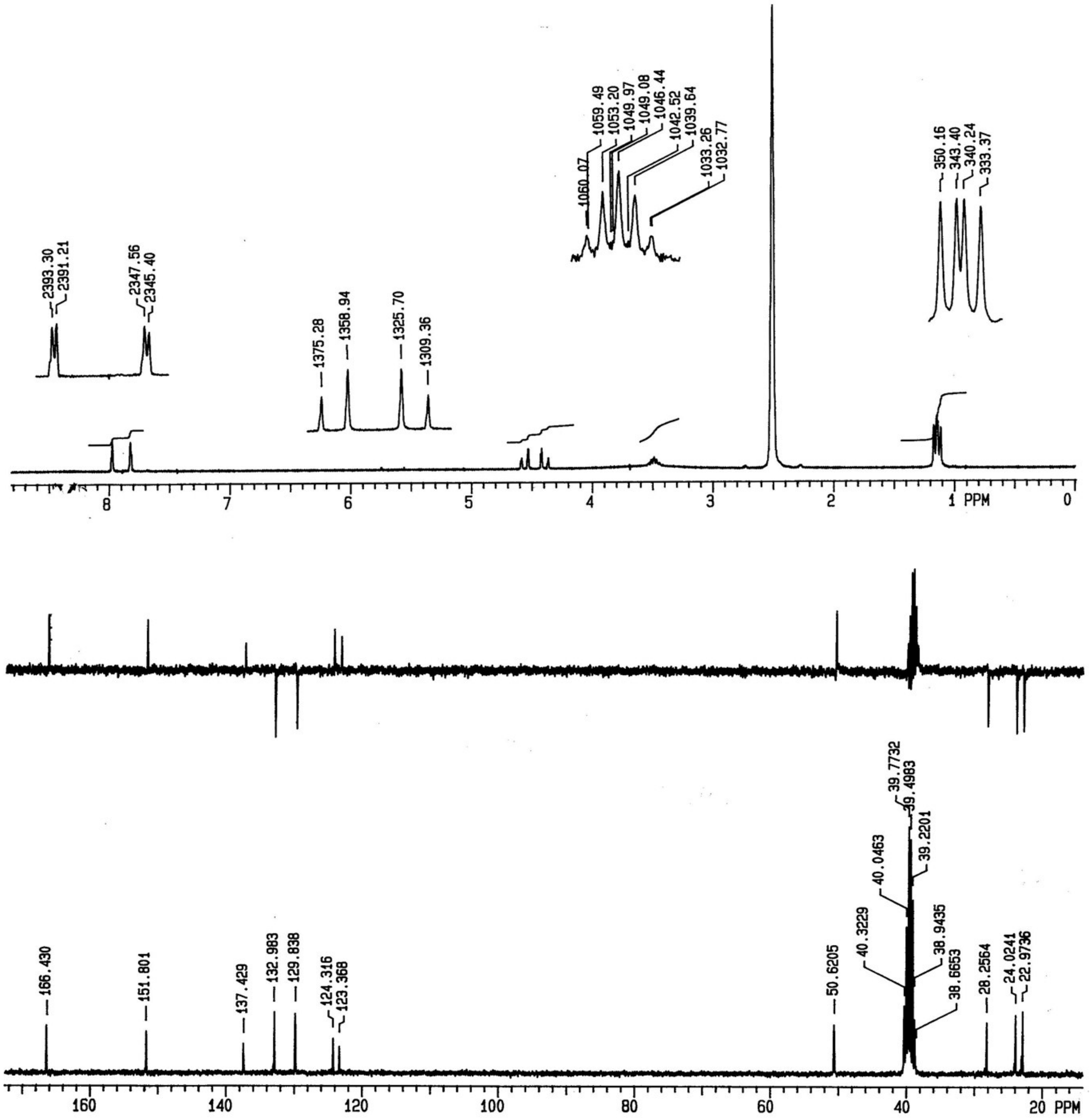

Figure 2S. ^{1}H NMR and ^{13}C NMR of ***E*-2c** in DMSO-d_6

3. X-ray crystalography

X-ray diffraction measurements for **2** and **4** were carried out with an Oxford Diffraction SuperNova diffractometer using hi-flux micro-focus Nova MoKα radiation. The unit cell determination and data integration were carried out using the CrysAlis package of Oxford Diffraction.[1] The structures were solved by direct methods using Olex2[2] software with the SHELXS structure solution program and refined by full-matrix

least-squares on F^2 with SHELXL-97[3] using an anisotropic model for non-hydrogen atoms. All H atoms were introduced in idealised positions (d_{CH} = 0.96 Å) using the riding model. The positional parameters for H-atoms attached to O were verified by the geometric parameters of the possible hydrogen bonds. The molecular plots were obtained using the Olex2 program. The crystallographic data and refinement details are quoted in Tables 1. CCDC (**2c**) 1849326, CCDC - 1849327 (**3c**) and CCDC - 1849329 (**6b**) contain the supplementary crystallographic data for this contribution. These data can be obtained free of charge via www.ccdc.cam.ac.uk/conts/retrieving.html (or from the Cambridge Crystallographic Data Centre, 12 Union Road, Cambridge CB2 1EZ, UK; fax: (+44) 1223-336-033; or deposit@ccdc.ca.ac.uk).

Table 1S. Crystal data and details of data collection.

Compound	**2c**	**3c**	**6b**
empirical formula	$C_{11}H_{12}Br_2N_2O_3$	$C_{11}H_{10}Br_2N_2O_2$	$C_{16}H_{17}BrN_2O_4$
Fw	380.56	362.03	381.22
T [K]	293	293	293
space group	$P2_1/c$	$P2_1/c$	$P2_1/c$
a [Å]	8.6989(11)	9.0273(13)	8.1295(6)
b [Å]	10.7694(15)	9.0598(6)	12.3462(8)
c [Å]	15.0669(17)	16.3781(16)	16.9591(7)
α [°]	90	90	90
β [°]	99.867(12)	100.771(13)	98.285(5)
γ [°]	90	90	90
V [Å^3]	1390.6(3)	1315.9(3)	1684.41(17)
Z	4	4	4
ρ_{calcd} [g cm^{-3}]	1.818	1.827	1.503
μ [mm^{-1}]	5.828	6.152	2.461
Crystal size [mm]	0.35 × 0.35 × 0.28	0.35 × 0.22 × 0.22	0.2 × 0.15 × 0.15
2Θ range	6.322 to 50.052	6.428 to 50.042	6.236 to 50.05
Reflections collected	9371	8750	12209
Independent	2447 [R_{int}= 0.1378]	2311 [R_{int}= 0.0527]	2969[R_{int}= 0.0434]
Data/restraints/parame	2447/0/166	2311/6/156	2969/0/212
R_1[a]	0.0750	0.0385	0.0387
wR_2[b]	0.1788	0.0786	0.0955
GOF[c]	1.007	1.043	1.058
Largest diff. peak/hole	0.63/-0.99	0.41/-0.4	0.40/-0.58

[a] $R_1 = \Sigma||F_o| - |F_c||/\Sigma|F_o|$. [b] $wR_2 = \{\Sigma[w(F_o^2 - F_c^2)^2]/\Sigma[w(F_o^2)^2]\}^{1/2}$. [c] GOF = $\{\Sigma[w(F_o^2 - F_c^2)^2]/(n - p)\}^{1/2}$, where n is the number of reflections and p is the total number of parameters refined.

Table 2S. Bond (Å) distances and angles (°) for the compounds **2c**, **3c** and **6b**

Compound **2c**

Br2-C3	1.876(7)	C7-C9	1.537(10)
Br1-C1	1.890(8)	O2-C11	1.213(9)
O1-C11	1.303(9)	C1-C2	1.366(11)
N2-N1	1.334(9)	C1-C6	1.369(11)
N2-O3	1.223(8)	C2-C3	1.391(10)
N1-C4	1.437(8)	C4-C5	1.375(10)
N1-C10	1.440(9)	C4-C3	1.391(10)
C7-C5	1.520(10)	C5-C6	1.405(10)
C7-C8	1.518(11)	C10-C11	1.494(11)
O3-N2-N1	112.9(7)	C3-C4-N1	118.3(7)
N2-N1-C4	116.2(6)	C4-C5-C7	123.6(7)
N2-N1-C10	121.4(6)	C4-C5-C6	118.1(7)
C4-N1-C10	122.3(6)	C6-C5-C7	118.3(7)
C5-C7-C9	112.2(6)	N1-C10-C11	113.9(7)
C8-C7-C5	111.3(7)	O1-C11-C10	112.0(7)
C8-C7-C9	111.2(7)	O2-C11-O1	124.5(8)
C2-C1-Br1	118.2(6)	O2-C11-C10	123.2(7)
C2-C1-C6	122.0(8)	C2-C3-Br2	117.9(6)
C6-C1-Br1	119.8(7)	C2-C3-C4	120.7(7)
C1-C2-C3	118.1(8)	C4-C3-Br2	121.4(6)
C5-C4-N1	121.0(7)	C1-C6-C5	120.3(8)
C5-C4-C3	120.7(7)		

Compound **3c**

Br1-C1	1.892(3)	C1-C6	1.383(5)
Br2-C3	1.884(4)	C2-C3	1.374(4)
O1-N2	1.374(4)	C3-C4	1.385(5)
O1-C11	1.410(5)	C4-C5	1.382(5)

O2-C11	1.215(4)	C5-C6	1.402(5)
N1-N2	1.304(4)	C5-C7	1.526(5)
N1-C4	1.446(4)	C7-C8	1.505(6)
N1-C10	1.328(4)	C7-C9	1.515(5)
C1-C2	1.374(5)	C10-C11	1.392(5)
N2-O1-C11	111.6(3)	C5-C4-N1	118.4(3)
N2-N1-C4	118.1(3)	C5-C4-C3	122.6(3)
N2-N1-C10	115.0(3)	C4-C5-C6	117.1(3)
C10-N1-C4	126.9(3)	C4-C5-C7	123.2(3)
N1-N2-O1	103.4(3)	C6-C5-C7	119.7(4)
C2-C1-Br1	118.1(3)	C1-C6-C5	119.5(4)
C2-C1-C6	122.8(3)	C8-C7-C5	110.5(3)
C6-C1-Br1	119.2(3)	C8-C7-C9	112.2(4)
C1-C2-C3	118.0(3)	C9-C7-C5	112.0(3)
C2-C3-Br2	118.3(3)	N1-C10-C11	107.5(4)
C2-C3-C4	120.0(4)	O2-C11-O1	118.9(4)
C4-C3-Br2	121.6(3)	O2-C11-C10	138.5(5)
C3-C4-N1	119.0(4)	C10-C11-O1	102.6(3)

Compound **6b**

Br1-C1	1.898(3)	C1-C6	1.372(4)
O13-C15	1.332(3)	C2-C3	1.379(4)
O13-C16	1.447(3)	C3-C4	1.379(4)
O15-C15	1.196(3)	C4-C5	1.386(4)
O17-C13	1.337(3)	C5-C6	1.397(4)
O17-C14	1.440(4)	C5-C7	1.519(4)
O19-C13	1.199(3)	C7-C8	1.530(5)
N1-N2	1.359(3)	C7-C9	1.523(5)
N1-C4	1.440(3)	C10-C11	1.414(4)
N1-C12	1.341(3)	C10-C15	1.484(4)
N2-C10	1.331(3)	C11-C12	1.373(4)

C1-C2	1.365(4)	C11-C13	1.475(4)
C15-O13-C16	116.1(2)	C1-C6-C5	120.4(3)
C13-O17-C14	116.3(2)	C5-C7-C8	110.2(3)
N2-N1-C4	118.6(2)	C5-C7-C9	111.2(3)
C12-N1-N2	112.3(2)	C9-C7-C8	110.8(3)
C12-N1-C4	129.1(2)	N2-C10-C11	111.4(2)
C10-N2-N1	104.52(19)	N2-C10-C15	118.9(2)
C2-C1-Br1	118.4(2)	C11-C10-C15	129.3(2)
C2-C1-C6	122.2(3)	C10-C11-C13	130.2(2)
C6-C1-Br1	119.4(2)	C12-C11-C10	104.3(2)
C1-C2-C3	118.5(3)	C12-C11-C13	125.3(2)
C2-C3-C4	119.9(3)	N1-C12-C11	107.5(2)
C3-C4-N1	116.6(2)	O17-C13-C11	110.1(2)
C3-C4-C5	122.3(2)	O19-C13-O17	124.3(3)
C5-C4-N1	121.0(2)	O19-C13-C11	125.6(3)
C4-C5-C6	116.8(2)	O13-C15-C10	111.6(2)
C4-C5-C7	122.7(2)	O15-C15-O13	124.4(3)
C6-C5-C7	120.5(3)	O15-C15-C10	124.0(2)

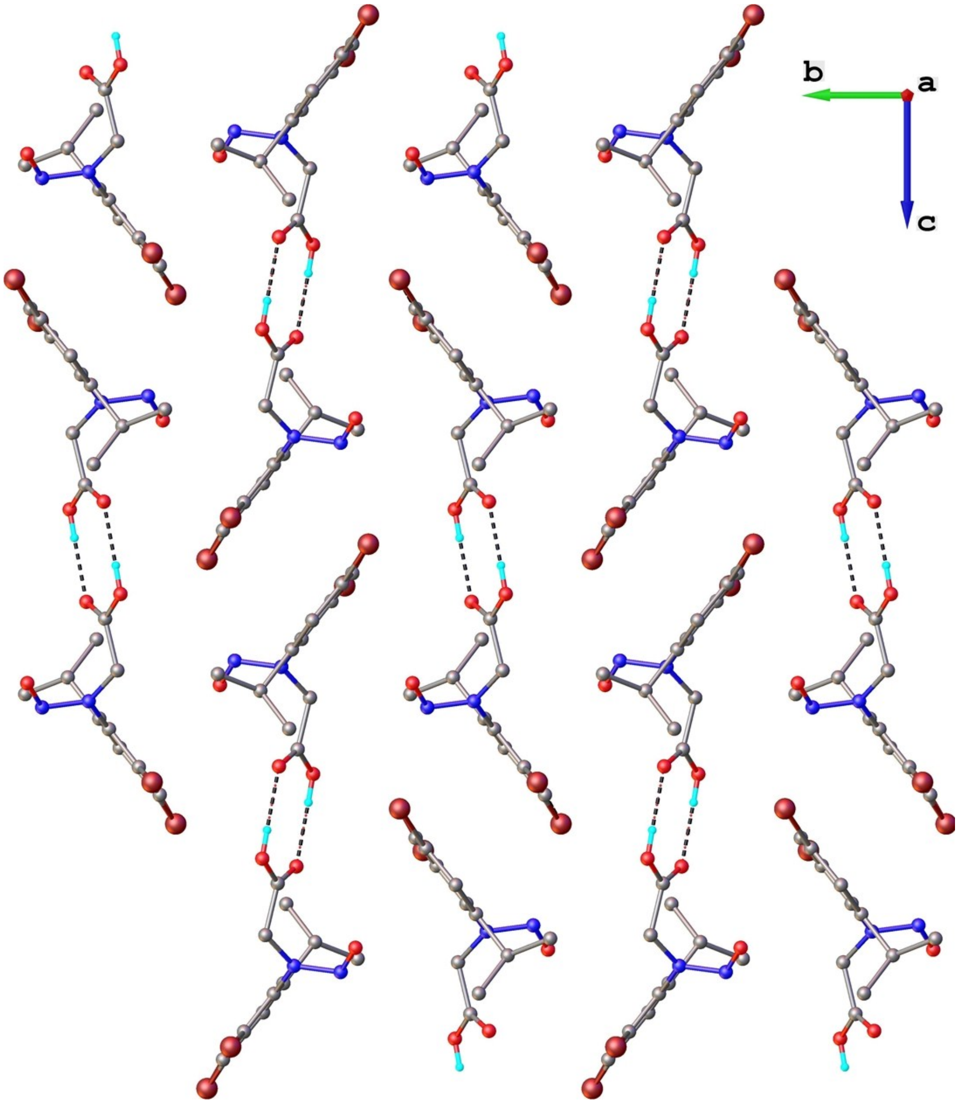

Figure 3S: Packing diagram of **2c** viewed along *a* axis. Non-relevant H-atoms are not shown. H-bonds are represented as dashed-black lines.

As could be seen, the central part of the supramolecular network features the formation of cyclic ring synthon of carboxylate dimer via two symmetrical C-H···O hydrogen bonds. The further association of the dimers to 1D network occurs through intermolecular Br···Br′ contacts at 3.536(1) Å.

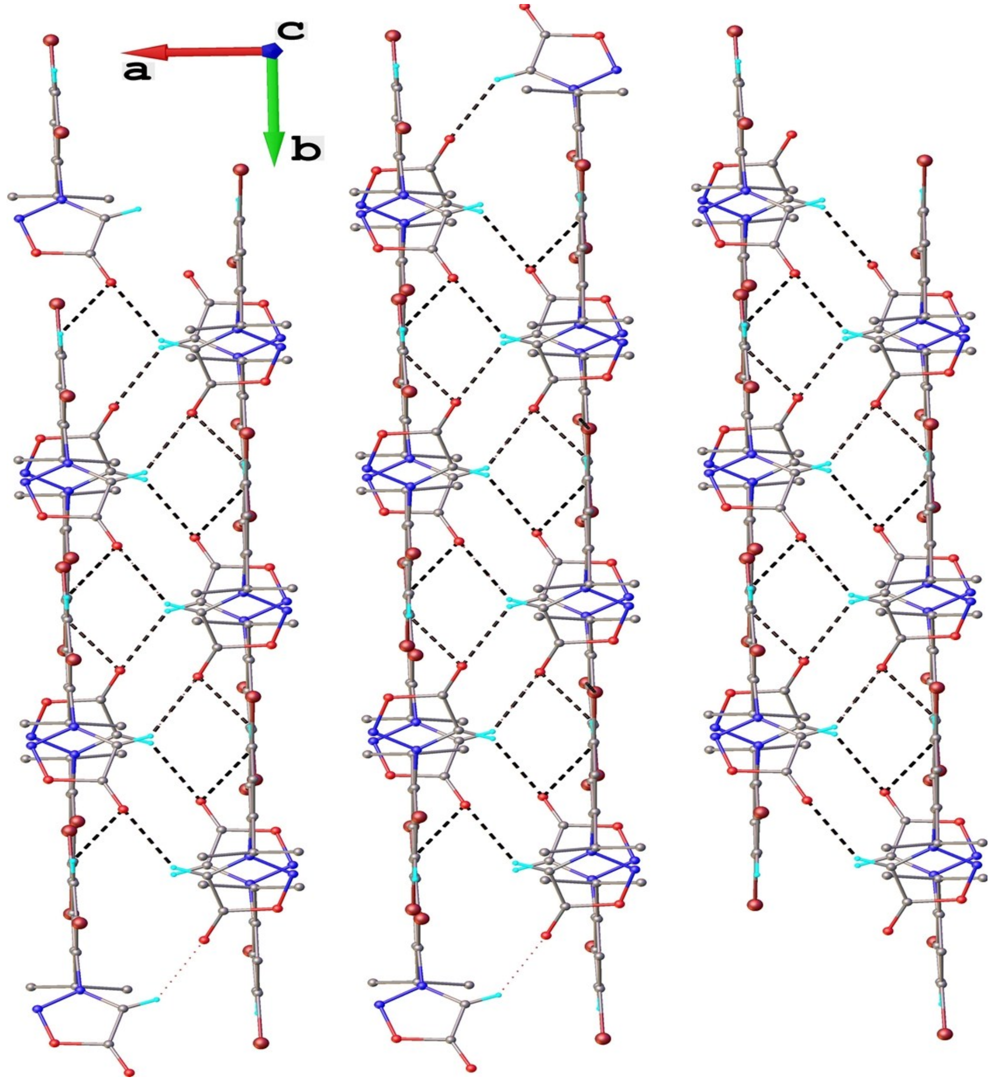

Figure 4S: Packing diagram of the crystal **3c** viewed along *c* crystallographic axis.

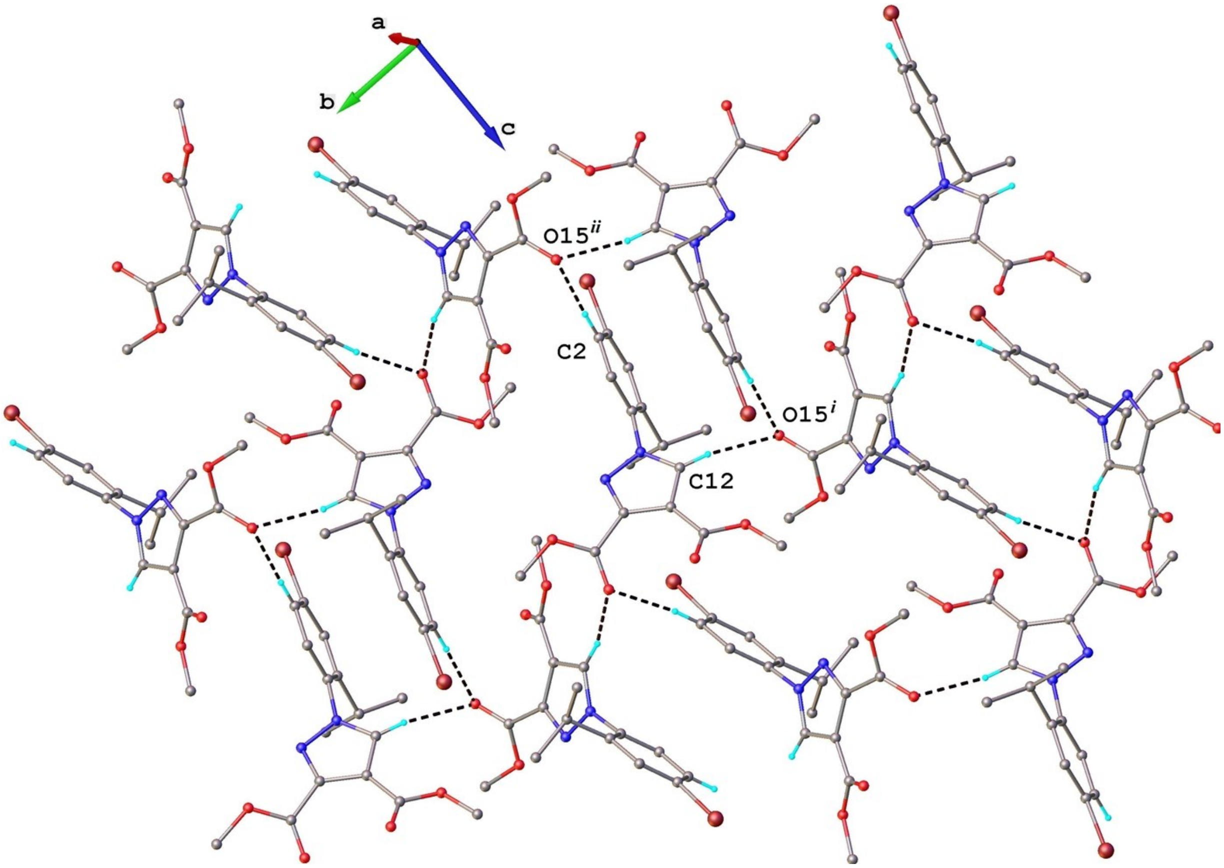

Figure 5S: 2D supramolecular network in the crystal structure **6b**. H-atoms not-involved in hydrogen bonding are omitted.C-H---O H- are shown in dashed-black lines. H-bond parameters: C12-H···O15 [C12-H 0.93 Å, H···O15 2.42 Å, C12···O15(1 - *x*, *y* – 0.5, 1.5 - *z*) 3.307(3) Å, ∠ C12HO15 158.5°]; C2-H···O15 [C2-H 0.93 Å, H···O15 2.39 Å, C2···O15(*x*, 1.5 - *y*, *z* – 0.5) 3.311(3) Å, ∠ C2HO15 169.8°].

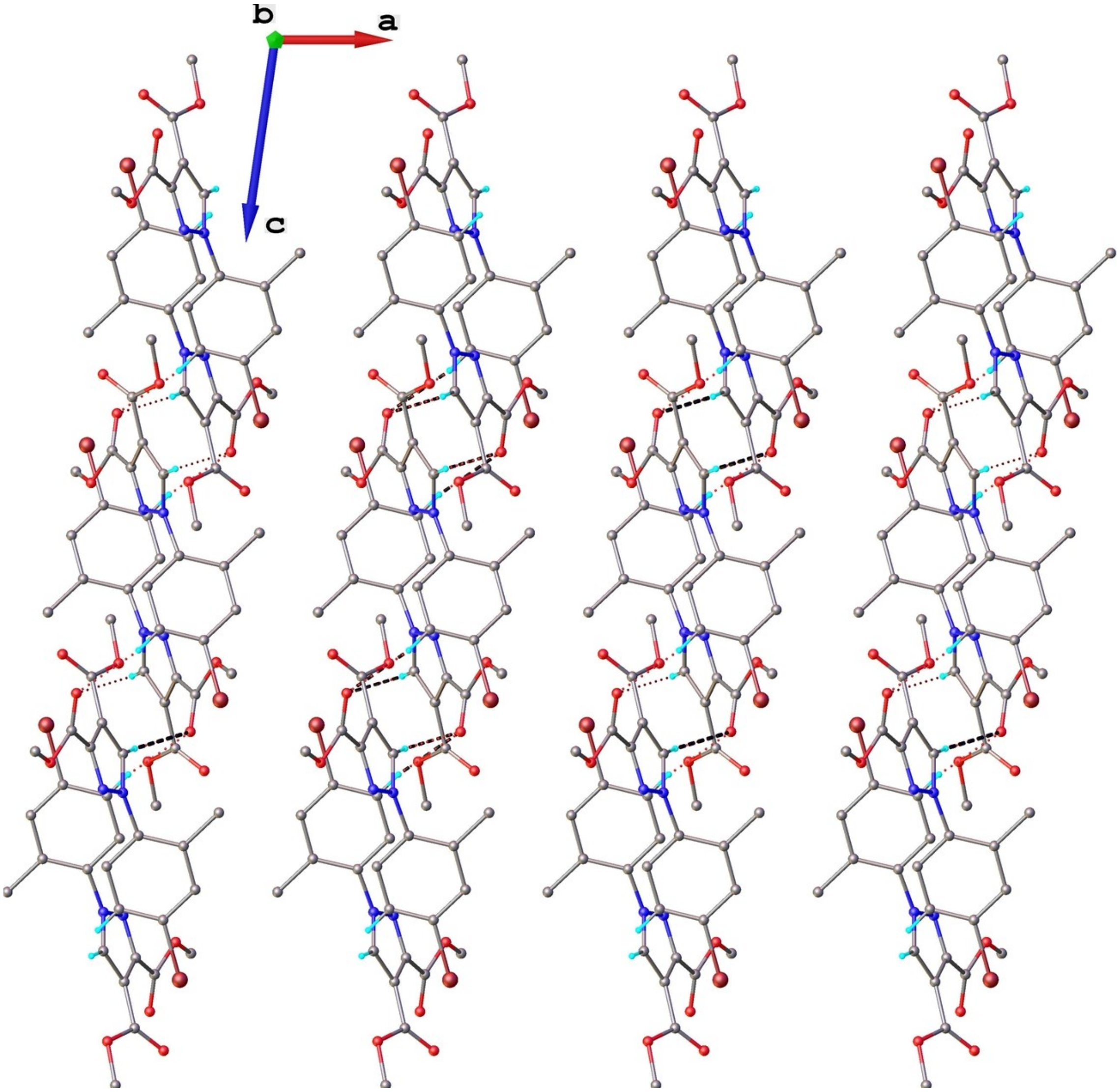

Figure 6S: Fragment of the crystal structure of **6b**, viewed along *b* axis. The methyl groups of isopropyl fragments are not shown for clarity.

1. CrysAlis RED, Oxford Diffraction Ltd.,Version 1.171.36.32, 2003

2. O. V. Dolomanov, L. J. Bourhis, R. J. Gildea, J. A. K. Howard, H. Puschmann, J. Appl. Cryst., 2009, 42, 339-341

3. G.M. Sheldrick, SHELXS, Acta Cryst., 2008, A64, 112-122.
